# Comparative description of ten transcriptomes of newly sequenced invertebrates and efficiency estimation of genomic sampling in non-model taxa

**DOI:** 10.1186/1742-9994-9-33

**Published:** 2012-11-29

**Authors:** Ana Riesgo, Sónia C S Andrade, Prashant P Sharma, Marta Novo, Alicia R Pérez-Porro, Varpu Vahtera, Vanessa L González, Gisele Y Kawauchi, Gonzalo Giribet

**Affiliations:** 1 Museum of Comparative Zoology, Department of Organismic and Evolutionary Biology, Harvard University, 26 Oxford Street, Cambridge, MA, 02138, USA; 2 Centro de Estudios Avanzados de Blanes, CSIC, c/ Accés a la Cala St. Francesc 14, Blanes, Girona, 17300, Spain; 3 Current address: Cardiff School of Biosciences, Cardiff University, BIOSI 1, Museum Avenue, Cardiff, CF10 3TL, UK; 4 Current address: Finnish Museum of Natural History, Zoology Unit, Pohjoinen Rautatiekatu 13, 00014 University of Helsinki, Helsinki, Finland

**Keywords:** Annelida, Arthropoda, Illumina, Mollusca, Nemertea, Next-generation sequencing, Porifera, Sipuncula

## Abstract

**Introduction:**

Traditionally, genomic or transcriptomic data have been restricted to a few model or emerging model organisms, and to a handful of species of medical and/or environmental importance. Next-generation sequencing techniques have the capability of yielding massive amounts of gene sequence data for virtually any species at a modest cost. Here we provide a comparative analysis of *de novo* assembled transcriptomic data for ten non-model species of previously understudied animal taxa.

**Results:**

cDNA libraries of ten species belonging to five animal phyla (2 Annelida [including Sipuncula], 2 Arthropoda, 2 Mollusca, 2 Nemertea, and 2 Porifera) were sequenced in different batches with an Illumina Genome Analyzer II (read length 100 or 150 bp), rendering between *ca*. 25 and 52 million reads per species. Read thinning, trimming, and *de novo* assembly were performed under different parameters to optimize output. Between 67,423 and 207,559 contigs were obtained across the ten species, post-optimization. Of those, 9,069 to 25,681 contigs retrieved blast hits against the NCBI non-redundant database, and approximately 50% of these were assigned with Gene Ontology terms, covering all major categories, and with similar percentages in all species. Local blasts against our datasets, using selected genes from major signaling pathways and housekeeping genes, revealed high efficiency in gene recovery compared to available genomes of closely related species. Intriguingly, our transcriptomic datasets detected multiple paralogues in all phyla and in nearly all gene pathways, including housekeeping genes that are traditionally used in phylogenetic applications for their purported single-copy nature.

**Conclusions:**

We generated the first study of comparative transcriptomics across multiple animal phyla (comparing two species per phylum in most cases), established the first Illumina-based transcriptomic datasets for sponge, nemertean, and sipunculan species, and generated a tractable catalogue of annotated genes (or gene fragments) and protein families for ten newly sequenced non-model organisms, some of commercial importance (i.e., *Octopus vulgaris*). These comprehensive sets of genes can be readily used for phylogenetic analysis, gene expression profiling, developmental analysis, and can also be a powerful resource for gene discovery. The characterization of the transcriptomes of such a diverse array of animal species permitted the comparison of sequencing depth, functional annotation, and efficiency of genomic sampling using the same pipelines, which proved to be similar for all considered species. In addition, the datasets revealed their potential as a resource for paralogue detection, a recurrent concern in various aspects of biological inquiry, including phylogenetics, molecular evolution, development, and cellular biochemistry.

## Background

Genetic studies in non-model organisms have been hindered by the lack of reference genomes, necessitating researchers to adopt time consuming and/or expensive experimental approaches. The advent of next-generation sequencing platforms (e.g., 454, Illumina, and SOLID), with concomitant decreases in sequencing costs due to escalating technological development, has made genomic and transcriptomic data increasingly accessible to research groups. To date, most *de novo* transcriptomes have been generated using Roche/454 (e.g. [1-5]) and have focused on single species. More recently, Illumina short reads have been used to build transcriptomic datasets in non-model species [6-11], or combined with 454 data to assemble whole genomes [12], offering promising prospects for the availability of such data for taxa of biological significance.

The advantages of transcriptomic data over genome sequencing range from their tractable size (ten to hundred times smaller than genomes) to their rapid procurement via large numbers of reads (from tens to a few hundred millions of short reads per lane, 100–150 bp) to facile assembly with intuitive software [13-15]. Transcriptomic sequencing offers advantages in the detection of rare transcripts with regulatory roles, given the enormous amount of reads covering each base pair (from 100 to 1,000x/bp generally) [16]. Also, transcriptomes contain fewer repetitive elements than genomes, reducing analytical burden during post-sequencing assembly. *De novo* assembled transcriptomes have been employed for gene discovery [3], phylogenomic analysis (e.g., [8,11,17-19]), microRNA and piRNA detection [16], detecting selection in closely related species [20], as well as for studies of differential gene expression (e.g. [2,7,21-23]), among other applications. Disadvantages of using transcriptomes for *de novo* assembly include issues with gene duplication, genetic polymorphism, alternative splicing, and transcription noise (e.g. [24,25]).

Many invertebrate phyla have been overlooked for genome and transcriptome sequencing priority, and for some groups, genomic data are particularly scarce. Among them, sponges (Porifera), ribbon worms (Nemertea), and peanut and segmented worms (Annelida) are particularly poorly studied with regard to genomics. The significance of such taxa stems from their utility for investigation of fundamental questions in evolutionary biology, such as the origins of metazoan organogenesis (e.g. [26], the evolution and loss of segmentation (e.g. [27-29]), and the evolution of terrestriality [30,31]. Lack of genomic data for these lineages is often accompanied by poor knowledge of basal relationships and evolutionary history. Furthermore, currently available genomic resources are often insufficient for studying a broad diversity of organisms, given the phylogenetic distance between the lineage of interest and the available model organisms. For example, among arthropods, traditional model organisms are restricted to Holometabola—the lineage of insects with complete metamorphosis—although many questions of evolutionary significance involve lineages outside of this derived group, such as the origin of flight at the base of Palaeoptera, and the evolution of terrestriality at the base of Hexapoda.

A comparative characterization of transcriptomic data across phyla in non-model species has not been carried out yet, and would be desirable for two reasons. First, generating such data enables estimating the efficacy of short-read data in sampling gene transcripts among distantly related lineages and with genomes of variable size. To date, Illumina data for comparative biology of multiple species have only been published for a few groups [8,11,32], but little has been done to compare libraries across different phyla. Second, this characterization is anticipated to guide future efforts to obtain transcriptomic data for non-model metazoans lineages, particularly those for which such efforts have not been previously undertaken. To abet forthcoming studies of development, phylogenomics, molecular evolution, and toxicology—among other applications of interest to us—we report here *de novo* assembled transcriptomes from 10 non-model invertebrate species belonging to five animal phyla: Porifera (*Petrosia ficiformis*, *Crella elegans*), Nemertea (*Cephalothrix hongkongiensis*, *Cerebratulus marginatus*), Annelida (*Hormogaster samnitica*, *Sipunculus nudus*), Mollusca (*Chiton olivaceus*, *Octopus vulgaris*) and Arthropoda (*Metasiro americanus*, *Alipes grandidieri*). Two species per phylum were selected (we grouped the annelid and the sipunculan species for comparison; although the relationships between these lineages are not well established, most studies favor either a sister relationship of the two or a paraphyletic Annelida that includes Sipuncula [18,29,33,34]) to allow comparisons within and among phyla. Among the species selected, one is important for fisheries (the common octopus, *Octopus vulgaris*) and another has medical significance due to its potent venom (e.g., the African centipede *Alipes grandidieri*).

In this article we characterized the effectiveness of the Illumina platform transcriptome sequencing strategy across these selected species with respect to data yield and quality. We compared the completeness of the datasets obtained for each taxon by assessing the sequencing depth and recovery of gene ontology identifications, as well as protein families. Also, searches of targeted genes (e.g., elements of conserved signaling pathways as well as housekeeping genes) in our datasets and their counterparts in three fully sequenced invertebrate genomes were used to compare and assess the suitability of our transcriptome datasets for gene discovery. Our study should thus contribute towards assessing the use of Illumina sequencing for *de novo* transcriptome assembly in non-model organisms as a cost-effective and efficient way to obtain vast amounts of comparable data for application in a broad array of downstream procedures.

## Results and discussion

### Transcriptome analysis

#### Assembling reads and selecting optimal assemblies

**Figure 1:**
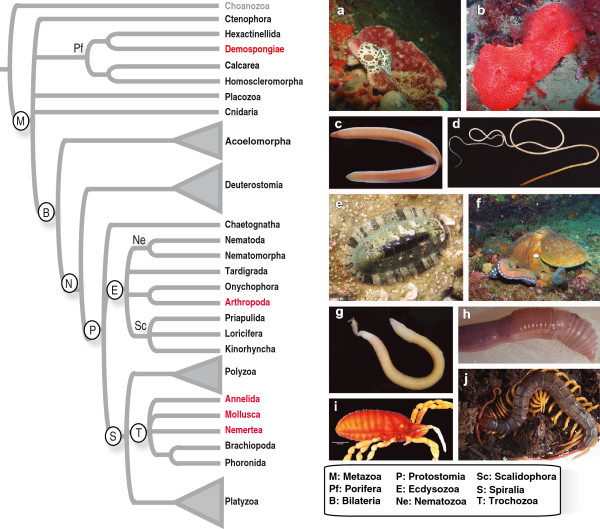
**Phylogenetic position of the higher taxonomic ranks of the species selected for this study, and accessory pictures of the living animals.a.***Petrosia ficiformis*. **b.***Crella elegans*. **c.***Cerebratulus marginatus*. **d.***Cephalothrix hongkongiensis*. **e.***Chiton olivaceus*. **f.***Octopus vulgaris*. **g**. *Sipunculus nudus*. **h**. *Hormogaster samnitica*. **i**. *Metasiro americanus*. **j**. *Alipes grandidieri*. (Pictures taken by Ana Riesgo (**a**), Alicia R. Pérez-Porro (**b**), Gonzalo Giribet (**c, f, j**), Sichun Sun (**d**), Jiri Nóvak (**e**), Gisele Kawauchi (**g**), Marta Novo (**h**), and Prashant Sharma (**i**).

cDNA libraries were obtained from high quality mRNA (Additional file 1) for the ten species (Figure 1) and yielded between *ca*. 25 and 52 million short reads using Illumina GAII (Table 1 and Additional file 2). After adaptor removal, thinning and trimming, we were left with *ca*. 15 to 45 million high quality reads per species, which were assembled using *de novo* assembly algorithms (Table 2 and Additional file 2). *De novo* assembly of either genomic or transcriptomic data poses substantial computational challenges [16,35,36]. Several short-read assemblers are now available, such as Velvet [13], ABySS [14], Trinity [36], and CLC Genomics Workbench (CLCbio, Aarhus, Denmark), among others. Most of these use de Bruijn graphs to assemble the reads, although there are slight variations among them, with few showing more efficiency [9,16,37-40]. We selected CLC for its desktop application with a graphical user-interface, which facilitates analysis of the transcriptomic data.

**Table 1:** Collecting information for the 10 species used for this study

**Phylum**	**Species**	**Class**, **Order**	**Collection site**	**Voucher number**	**Body part**	**Preservation**
Porifera	*Petrosia ficiformis*	Demospongiae, Haplosclerida	Punta Santa Anna, Blanes, Girona, Spain	DNA105722*	Entire animal	LN_2_/-80°C
	*Crella elegans*	Demospongiae, Poecilosclerida	Tossa de Mar, Girona, Spain	DNA105740*	Entire animal	RNA*later*
Nemertea	*Cephalothrix hongkongiensis*	Anopla, Paleonemertea	Akkeshi, Hokkaido, Japan	DNA106145*	Entire animal	RNAlater
	*Cerebratulus marginatus*	Anopla, Heteronemertea	False Bay, San Juan Island, Washington, USA	DNA105590*	Entire animal	LN_2_/-80°C
Mollusca	*Chiton olivaceus*	Polyplacophora, Chitonida	Tossa de Mar, Girona, Spain	DNA106012*	Entire animal	RNA*later*
	*Octopus vulgaris*	Cephalopoda, Octopoda	Blanes Bay, Blanes, Girona, Spain	DNA106283*	Fragment of arm	RNA*later*
Sipuncula	*Sipunculus nudus*	Sipunculidae	Fort Pierce, Florida, USA	DNA106878*	Distal fragment of animal	LN_2_/-80°C
Annelida	*Hormogaster samnitica*	Oligochaeta, Opisthopora	Gello, Toscana, Italy	GEL6**	Distal fragment of animal	RNA*later*
Arthropoda	*Metasiro americanus*	Arachnida, Opiliones	Kingfisher Pond, Savannah, Georgia, USA	DNA101532*	Entire animal	LN_2_/-80°C
	*Alipes grandidieri*	Chilopoda, Scolopendromorpha	Tanzania; pet supplier (http://www.kenthebugguy.com)	DNA106771*	Mid part of body	LN_2_/-80°C

Voucher numbers refer to specimens collected in the same area as the one used for the nucleic extraction, since most of the times the entire animal (or the entire collected piece of animal) was processed. A single asterisk refers to voucher numbers in the Museum of Comparative Zoology, Harvard University, and a double asterisk to those deposited in the Department of Zoology and Physical Anthropology, Universidad Complutense de Madrid. In all cases only one specimen was used for extraction, except for *Metasiro americanus*, which also had embryos in several developmental stages.

**Table 2:** Assembly parameters

	**N reads BT**	**N reads AT**	**%reads discarded**	**Avg.L AT**	**NRMC**	**N contigs**	**N bases(Mb)**	**Avg. L Contigs**	**SD**	**Maximum Contig Length(bp)**	**N50**	**Avg.L**	**SD**
***Petrosia ficiformis***	49,758,556	32,612,454*	34.5	65.4	28,439,277	67,423	29.9	443.3	370.7	7,377	503	926.8	496.6
***Crella elegans***	26,513,534	25,951,906*	2.1	93.1	16,464,495	71,524	26.7	372.7	261.7	4,637	437	682.1	333.1
***Cephalothrix hongkoiensis***	51,091,244	26,631,980*	47.9	79.8	14,447,555	76,507	28.8	376.7	242.7	5,198	390	652.8	300.1
***Cerebratulus marginatus***	51,711,276	46,967,592*	9.2	73.8	22,977,409	109,947	57.1	518.0	394.2	7,731	559	991.0	521.6
***Chiton olivaceus***	46,265,184	40,889,060*	11.6	98.5	32,085,523	207,559	75.9	366.0	238.6	9,374	372	627.0	305.3
***Octopus vulgaris***	16,431,468	15,422,631*	6.1	125.0	11,670,780	77,383	41.7	540.0	125.0	16,472	599	1122.9	660.5
***Sipunculus nudus***	45,973,825	43,842,184**	4.6	100.5	25,679,520	71,960	31.2	431.7	228.0	3,032	437	676.2	262.5
***Hormogaster samnitica***	50,789,952	47,857,894**	5.8	96.5	32,511,666	190,189	75.9	399.8	312.5	7,319	423	766.6	426.8
***Metasiro americanus***	24,943,641	23,959,711**	3.9	129.6	19,735,275	101,929	43.9	439.5	423.0	10,407	477	1,010.3	621.7
***Alipes grandidieri***	32,294,430	31,561,359**	2.3	134.8	25,457,734	162,326	59.9	380.9	306.9	9,323	377	710.7	443.4

Grey background indicates libraries sequenced for 150 bp; otherwise they are 100 bp. Abbreviations: *N*, number; *BT*, before thinning and trimming; *AT*, after thinning and trimming; *NMRC*, number of reads matched to contigs; *Mb*, megabases; *bp*, base pairs; *avg*., average; *L*, length; *SD*, standard deviation; *, thinning limit of 0.05; **, thinning limit of 0.005.

**Figure 2:**
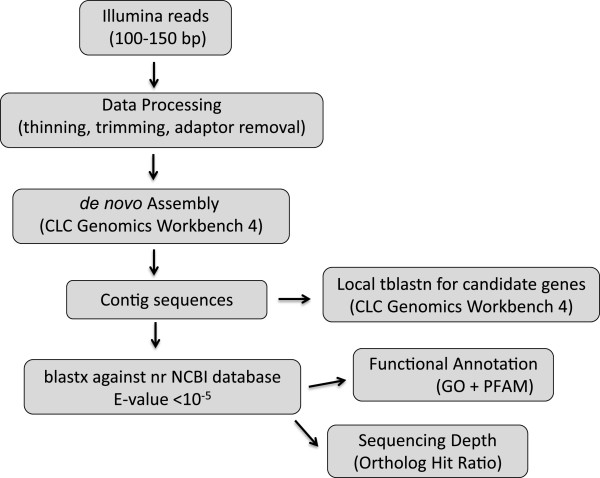
Workflow followed for the transcriptome analysis.

We processed the sequences obtained following the workflow shown in Figure 2. The filtering of reads based on quality parameters when using 0.005 as the limit resulted in removal of a larger portion of each read when low quality was detected, and in many instances an entire low-quality read was removed. Trimming performed with 0.005 as the limit was preferred if the initial quality of the reads was not very high. Otherwise, the least stringent value was preferred. Mean length of reads ranged between 65.4 bp in *Petrosia ficiformis* to 134.8 bp for *Alipes grandidieri* (Additional file 2). Although one may expect to have longer contigs with higher numbers of reads (Table 2), contig size did not have a direct correlation with the number of input reads. The length of the reads used for the assembly appeared to have an effect on the length of the assembled contigs—the longest contigs appearing when the read length was greater than 120 bp (Table 2 and Additional files 2 and 3). Assemblies performed with reads originally sequenced at 101 bp had an average maximum contig length of 6,939 bp ± 1,744.9 bp, whereas those obtained with reads originally sequenced at 150 bp showed larger numbers (9,809 ± 5,505.1 bp) of longest contigs.

Among the two resulting assemblies for each species (A and B, see Methods section; Additional file 2), we selected one (Table 2) based on combinations of optimality criteria (Additional file 4). The assemblies performed with the largest numbers of reads were not always the optimal ones (see Table 2 and Additional file 2). Parameters that affected the final decision were: number of contigs, number of bases, N50, number of contigs longer than 2 Kb, and maximum contig length (Additional file 4). In all cases, the selected assembly was that containing the largest amount of contigs over 2 Kb (Additional file 2). Only the selected assemblies are discussed below (Table 2 and Additional file 2).

#### *Transcriptome descriptors*: *number and length of contigs*

More than 40% of the reads were successfully assembled into contigs in all cases (Table 2), with more than 85% of the reads matching to resulting contigs in *P*. *ficiformis* (Table 2). Coverage values for our transcriptomes (defined by number of reads covering a single base in each contig) varied between the lowest value of 36.2 in *Cerebratulus marginatus* to the highest value of 92.1 in *Sipunculus nudus* (see Table 3). In all cases, the longer the contig, the higher the coverage for each base (Additional file 5), although in some cases such as *Chiton olivaceus* and *Sipunculus nudus*, coverage values were much higher in shorter contigs (Additional file 5). Coverage values are usually higher for Illumina than for other NGS platforms, ranging from around 5 to 7 for 454 datasets [1,41,42], to more than 30 for Illumina [9,39,43]. The average number of reads building each contig varied greatly, ranging from 421.7 reads for *Petrosia ficiformis* to 124.3 reads for *Chiton olivaceus* (see Table 3). The maximum number of reads used to build each contig ranged from 65,985 in *Octopus vulgaris* to 543,848 in *Hormogaster samnitica*, and the minimum of 1 or 2 reads for each species (Table 3). Since very short contigs could be built with 1 paired-end read, we removed all contigs below 300 bp for each species prior to subsequent analyses. The minimum coverage for the sub-selections was highly variable: between 2 and 10 reads per contig (see Table 3). Our coverage results suggested the possibility of redundancy in the sequencing process (i.e., a great number of reads assembling into one contig, meaning a much deeper sequencing of some DNA fragments). This redundancy was tolerated because the downstream applications for these datasets, include gene expression and/or population genetics, for which redundancy can be addressed at a later analytical step [44].

**Table 3:** Coverage for the selected assemblies per species, estimated as the number of reads per bp and number of reads used to build the contigs (average value and maximum and minimum values)

	**Reads**/**bp**		**N reads forming the contigs**			
	**Average**	**Max. Avg. Cov. (length contig bp)**	**Average**	**Min. N**	**Max. N**	**Min. N reads contigs** >**300bp**
***Petrosia ficiformis***	64.7	31926.9 (309)	421.7	2	113,180	9
***Crella elegans***	72.7	88692.0 (238)	230.2	2	317,465	5
***Cephalothrix hongkongiensis***	48.7	74756.8 (337)	172.5	2	173,829	6
***Cerebratulus marginatus***	36.2	56724.0 (657)	208.9	2	307,273	5
***Chiton olivaceus***	45.2	91002.5 (217)	124.3	2	168,082	3
***Octopus vulgaris***	38.4	27963.1 (490)	151.0	2	65,985	3
***Sipunculus nudus***	92.1	123567.7 (463)	355.0	2	412,174	10
***Hormogaster samnitica***	40.6	85181.4 (273)	171.3	2	543,848	3
***Metasiro americanus***	61.3	58777.3 (201)	186.2	1	89,980	2
***Alipes grandidieri***	65.3	98893.9 (211)	161.8	2	153.215	2

Also, the minimum number of reads used to build the contigs longer than 300 bp is given. *N*, number; *SD*, standard deviation, *bp*, base pairs.

An average of 47.1 Mb (ranging from 26.7 for *Crella elegans* to 75.9 Mb for *Chiton olivaceus* and *Hormogaster samnitica*; Table 2) were assembled into contigs in our datasets, with results falling in a range comparable to other previous studies with non-model species using 454 [41,45], although in many cases the assemblies were smaller [1]. Likewise, prior assemblies performed with Illumina reads ranged from 20 to 30 Mb [24,43,46-48], values lower than ours, probably because they used shorter sequencing lengths.

Contig N50 is a weighted median statistic such that 50% of the entire assembly is contained in contigs equal to or larger than this value (in bp). N50 for a genome is usually around 1 Kb, which represents the average size of an exon for animals [49]. The lowest N50 recovered among our selected datasets was that of *Chiton olivaceus* (372, with an average length of 627.0 ± 305.3 bp) and the highest was for *Octopus vulgaris* (599, with an average length of 1,122.9 ± 660.5 bp) (see Table 2). These values are smaller than those observed for transcriptomes assembled from 454 pyrosequencing data (e.g., 900 bp for the chickpea [39]; 893 bp for *Oncopeltus*[41]; 693 bp for *Acropora*[1]) but similar to N50s obtained with Illumina RNAseq (e.g. [24,48]).

Our datasets contained a larger number of short contigs when compared to data obtained with 454 pyrosequencers (e.g. [2,4,50]), with only 4.7% to 15.7% of our assemblies constituted by contigs > 1 Kb (Additional file 3). However, the proportion of contigs over 1 Kb found in our data was surprisingly high for transcriptomic data (Additional files 2 and 6), surpassing that of 454 sequencing in other invertebrates with comparable sequencing effort, and similar to assemblies built with equal numbers of Illumina reads [8,46]. For instance, the transcriptome of the deep-sea mollusk *Bathymodiolus azoricus* (sequenced with 454) contained 3,071 contigs over 1 Kb [45], a smaller number than the > 5,000 contigs longer than 1 Kb in our mollusks, *Chiton olivaceus* and *Octopus vulgaris* (Additional file 6). Similarly, our results for arthropods (Additional file 6) outperform those obtained with 454 for several arthropod species [2,4,50]. Interestingly, our results for the number of contigs over 1 Kb (and also contigs > 500 bp) in the sponges *Petrosia ficiformis* and *Crella elegans* (Additional file 6) are similar to those found for the coral *Acropora millepora*, using 454 [22], indicating a similar sequencing depth.

#### Detection of chimeric sequences

The maximum contig length for each species varied greatly, ranging from 3,032 bp for *Sipunculus nudus*—the library with the lowest values for most metrics of data quality—to 16,472 bp for *Octopus vulgaris* (Table 2). The appearance of very long contigs in transcriptomic assemblies can be due to the existence of chimeric or miss-assembled sequences. Therefore, to check for putative chimeras (assembly artifacts), we translated the longest contig for each assembly to all 6 possible reading frames, took the longest open reading frame, and re-blasted it using the blastp program in NCBI. We also blasted the first and last 500 bases of each contig to check whether they recovered the same blast hit. For all assemblies, except for *Sipunculus nudus*, the longest contig translated to well-known proteins with e-values *ca*. 10^-5^ with both the beginning and end retrieving the same blast hits. The longest contigs corresponded to a protocadherin for *P*. *ficiformis*, an Ets DNA binding protein for *Crella elegans*, fibrillin 2 proteins for *C*. *marginatus* and *M*. *americanus*, a collagen type IV for *C*. *hongkongiensis*, an apolipophorin for *C*. *olivaceus*, titin for *O*. *vulgaris*, CCR4-NOT transcription complex for *H*. *samnitica*, and a low density lipid receptor-related protein for *A*. *grandidieri*. In the case of *S*. *nudus*, the two longest contigs contained small reading frames, while the third longest contig contained a sequence resembling a growth hormone inducible transmembrane protein. The success in sequencing a complete transcriptome is difficult to assess without a reference genome or without functional assays. Therefore, even though our transcriptome datasets did not show evidence of chimeric matching of reads, we cannot ascertain the overall sequencing success in terms of coverage of the corresponding genome. However, one of the advantages of the large sequencing depth generated by Illumina is that it ensures more complete and effective coverage of the transcriptomes [24,51] than that of 454, preventing the appearance of mismatched assemblies of reads from different genes. Overall, our results also indicate that the production of dozens of millions of reads with Illumina often provide more complete transcriptomic datasets at a lower cost than those obtained with 454 (which usually render less than 1 million reads). This has been recently shown in a study on mollusk phylogenomics [8], where matrix completeness for Illumina data is superior to 454 data, and comparable to the data for *Lottia gigantea*, for which a whole genome was available.

### Functional annotation

#### Gene ontology terms

**Figure 3:**
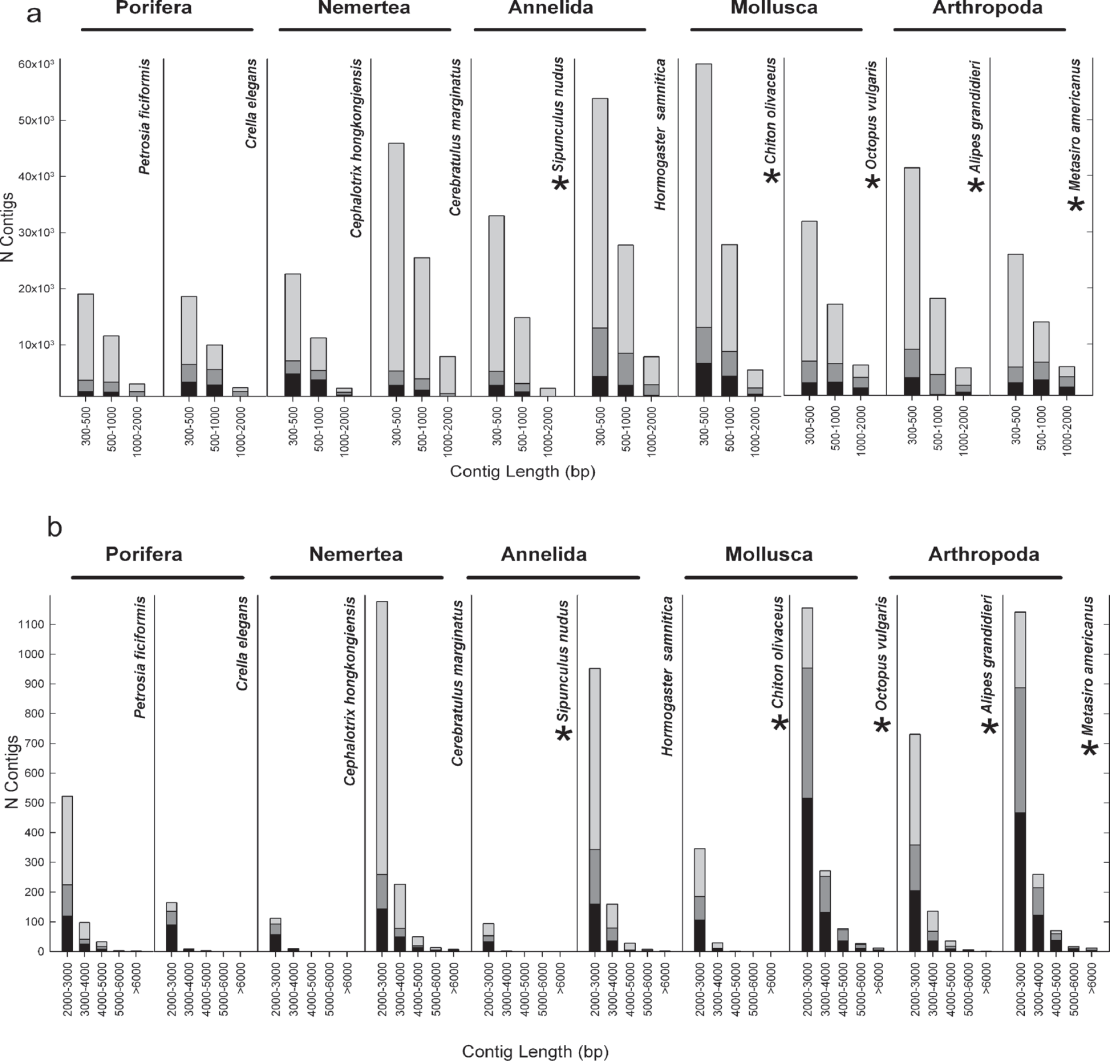
**Size distribution of a. short contigs(between 300 and 2,000 bp) and b. long contigs(from 2,001 to >6,000 bp) without blast hit (light grey), with blast hit (dark grey) and with annotation or GO assignment (black).** Asterisks represent species for which datasets were obtained using read length of 150 bp.

Contigs above 300 bp for each of the selected assemblies were blasted against a selection of the nr database (Metazoa + Fungi). Roughly between 9,000 and 26,000 transcripts per species recovered blast hits (Table 4 and Additional file 7), only half of these being annotated (i.e., with an assigned GO term) in each case (Table 4 and Additional file 7). These numbers are similar to those of previous studies with both animal [1,9,41,45,52,53] and plant [39,42,47,48]*de novo* assembled transcriptomes. When the frequencies of contigs with blast hits and annotations were plotted against contig size, it became obvious that the longest contigs yielded blast hits and annotations with a higher frequency (Figure 3). Very short contigs (300–500 bp) rarely returned blast hits or annotations, with approximately 60% to 90% of these sequences having an unidentifiable affiliation (Figure 3a). In nearly all transcriptomes, around 70% of the contigs between 2,000 and 3,000 bp retrieved blast hits and annotations (Figure 3b), (except in *Cerebratulus marginatus* and *Hormogaster samnitica*; 22% and 35%, respectively) (Figure 3b). In the case of the nemertean, this could be due to the lack of a closely related reference genome. For the longest contigs (more than 3,000 bp), the percentage of blasted or annotated contigs was always higher than 70% (Figure 3b). The total number of contigs annotated with BLAST2GO ranged between 4,942 in *S*. *nudus* and 12,533 in *C*. *olivaceus* (Table 4).

**Table 4:** Number of transcripts with blast hits and associated Gene Ontology (GO) terms for each transcriptome

	**N Contigs unidentified**	**N Contigs with Blast Hits**	**N Contigs with GOs**
***Petrosia ficiformis***	26,291	9,069	5,380
***Crella elegans***	17,719	13,984	7,288
***Cephalotrix hongkongiensis***	22,035	14,251	9,778
***Cerebratulus marginatus***	69,803	11,062	5,722
***Chiton olivaceus***	69,384	24,495	12,533
***Octopus vulgaris***	37,851	18,881	9,165
***Sipunculus nudus***	40,946	9,322	4,942
***Hormogaster samnitica***	65,247	25,681	8,806
***Metasiro americanus***	29,382	18,056	9,720
***Alipes grandidieri***	49,511	16,688	9,691

It should be noted that we are not considering all unique hits as individual genes, because transcriptomic assemblies can contain sequences belonging to non-overlapping fragments of the same gene. As a result, if a redundancy test is not performed, the number of unique blast hits found in transcriptomic data may be a gross overestimation of the number of genes present in the genomes of the focal taxa. We analyzed the level of redundancy in the blast searches (i.e., unique hits = only one contig matching each protein; redundant hits = more than one contig matching the same protein). *Crella elegans* showed the highest redundancy levels, with only 80.1% as unique hits, whereas *Cerebratulus marginatus* recovered 93.6% unique hits in the blast searches (Figure 3). Among the redundant hits, most of them were putative transposable elements (Table 5), which are known to comprise a large portion of genomes [54-56]. However, sequences of the metazoan transponsable elements are known for very few species [55], and therefore the occurrence of several hits to the same protein sequence could reflect lack of knowledge, rather than redundant sequencing or deficient assembly. Interestingly, none of the most redundant hits in *Hormogaster samnitica* was a transposable element (Table 5), and in this case the redundancy might be due to the occurrence of several splice variants of the same gene and non-overlapping fragments of the gene. In the case of the most redundant protein of *Cerebratulus marginatus*, the redundancy was caused by both factors in equal proportion: there were 3 paralogous sequences (or splice variants) that were fragmented. In both sponges, the most redundant hit corresponded to the putative eukaryotic initiation factor 4E of *Amphimedon queenslandica* (Table 5), which is a protein of *ca*. 42,000 amino acids, and thus the several contigs that matched it are fragments of the same gene that failed to be assembled.

**Table 5:** Protein names and lengths (in aminoacids, aa) for the five most redundant hits in each transcriptome

**# Hits**	**Protein name and[species name]**	**Putative transposable element**	**Protein length(aa)**	**Accession number**
***Petrosia ficiformis***				
x9	PREDICTED: hypothetical protein LOC100641198 [*Amphimedon queenslandica*]	-	673	XP_003382742
x9	PREDICTED: hypothetical protein LOC100639583 [*Amphimedon queenslandica*]	yes	1768	XP_003390293
x10	PREDICTED: RING finger protein 213-like [*Amphimedon queenslandica*]	-	5361	XP_003389786
x12	ankyrin 2,3/unc44 [*Aedes aegypti*]	-	789	XP_001649474
x16	PREDICTED: hypothetical protein LOC100637079 [*Amphimedon queenslandica*]	-	41943	XP_003386025
***Crella elegans***				
x25	Collagen protein [*Suberites domuncula*]	-	282	CAC81019
x36	aggregation factor protein 3, form C [*Microciona prolifera*]	-	2205	AAC33162
x38	PREDICTED: deleted in malignant brain tumors 1 protein-like [*Amphimedon queenslandica*]	-	3131	XP_003389240
x46	PREDICTED: hypothetical protein LOC100640736 [*Amphimedon queenslandica*]	-	5715	XP_003383871
x193	PREDICTED: hypothetical protein LOC100637079 [*Amphimedon queenslandica*]	-	41943	XP_003386025
***Cephalothrix hongkongiensis***				
x14	pol-like protein [*Ciona intestinalis*]	yes	1235	BAC82623
x14	pol-like protein [*Ciona intestinalis*]	yes	1263	BAC82626
x15	PREDICTED: similar to ORF2-encoded protein, partial [*Hydra magnipapillata*]	yes	372	XP_002155414
x15	PREDICTED: Pao retrotransposon peptidase family protein-like [*Saccoglossus kowalevskii*]	-	1559	XP_002731015
x23	putative zinc finger protein [*Schistosoma mansoni*]	-	486	CCD80531
***Cerebratulus marginatus***				
x9	PREDICTED: hypothetical protein LOC497165 [*Danio rerio*]	yes	2265	XP_003200870
x11	ORF2-encoded protein [*Danio rerio*]	yes	1027	BAE46429
x11	PREDICTED: similar to ORF2-encoded protein, partial [*Strongylocentrotus purpuratus*]	yes	1117	XP_001187755
x11	PREDICTED: similar to ORF2-encoded protein [*Strongylocentrotus purpuratus*]	yes	1124	XP_001189850
x11	PREDICTED: hypothetical protein LOC100535924 [*Danio rerio*]	-	1448	XP_003199942
***Octopus vulgaris***				
x38	PREDICTED: hypothetical protein LOC100609033 [*Pan troglodytes*]	yes	255	XP_003317434
x44	PREDICTED: hypothetical protein LOC100597269 [*Nomascus leucogenys*]	yes	220	XP_003276349
x57	PREDICTED: hypothetical protein LOC100414382, partial [*Callithrix jacchus*]	yes	178	XP_002762361
x57	PREDICTED: zinc finger protein 91-like [*Acyrthosiphon pisum*]	-	818	XP_003243211
x90	PREDICTED: hypothetical protein LOC100608502, partial [*Pan troglodytes*]	yes	211	XP_003315526
***Chiton olivaceus***				
x16	predicted protein [*Nematostella vectensis*]	yes	1079	XP_001630327
x17	PREDICTED: similar to tyrosine recombinase [*Strongylocentrotus purpuratus*]	-	461	XP_001183896
x22	pol-like protein [*Biomphalaria glabrata*]	yes	1222	ABN58714
x29	hypothetical protein EAI_13357 [*Harpegnathos saltator*]	-	172	EFN88744
x48	PREDICTED: similar to ORF2-encoded protein, partial [*Hydra magnipapillata*]	yes	372	XP_002155414
***Sipunculus nudus***				
x7	dopamine beta hydroxylase-like protein, partial [*Pomatoceros lamarckii*]	-	504	ADB11406
x7	pol-like protein [*Ciona intestinalis*]	yes	1263	BAC82626
x7	PREDICTED: similar to transposase [*Strongylocentrotus purpuratus*]	yes	1312	XP_001193486
x9	pol-like protein [*Ciona intestinalis*]	yes	1235	BAC82623
x11	lectin 1B [*Arenicola marina*]	-	243	ADO22714
***Hormogaster samnitica***				
x15	leechCAM [*Hirudo medicinalis*]	-	858	AAC47655
x15	pannexin 4 [*Aplysia californica*]	-	413	NP_001191576
x16	predicted protein [*Nematostella vectensis*]	-	2047	XP_001624963
x19	hypothetical protein CBG_27119 [*Caenorhabditis briggsae* AF16]	-	224	CAR99373
x24	tractin [*Hirudo medicinalis*]	-	1880	AAC47654
***Metasiro americanus***				
x14	transglutaminase [*Limulus polyphemus*]	-	764	2012342A
x15	putative reverse transcriptase [*Takifugu rubripes*]	yes	851	AAK58879
x30	hypothetical protein BRAFLDRAFT_210900 [*Branchiostoma floridae*]	-	489	XP_002611360
x39	hypothetical protein BRAFLDRAFT_79800 [*Branchiostoma floridae*]	-	512	XP_002597956
x53	hypothetical protein BRAFLDRAFT_89523 [*Branchiostoma floridae*]	-	396	XP_002590717
***Alipes grandidieri***				
x55	PREDICTED: similar to predicted protein [*Hydra magnipapillata*]	yes	1371	XP_002161911
x56	Transposable element Tcb1 transposase [*Salmo salar*]	yes	281	ACN11475
x57	hypothetical protein TcasGA2_TC002110 [*Tribolium castaneum*]	yes	346	EEZ99596
x58	hypothetical protein EAG_05969 [*Camponotus floridanus*]	yes	282	EFN71217
x123	hypothetical protein TcasGA2_TC000717 [*Tribolium castaneum*]	yes	346	EEZ98274

Their putative transposable element nature is indicated, as well as the Genbank accession number for each protein.

**Figure 4:**
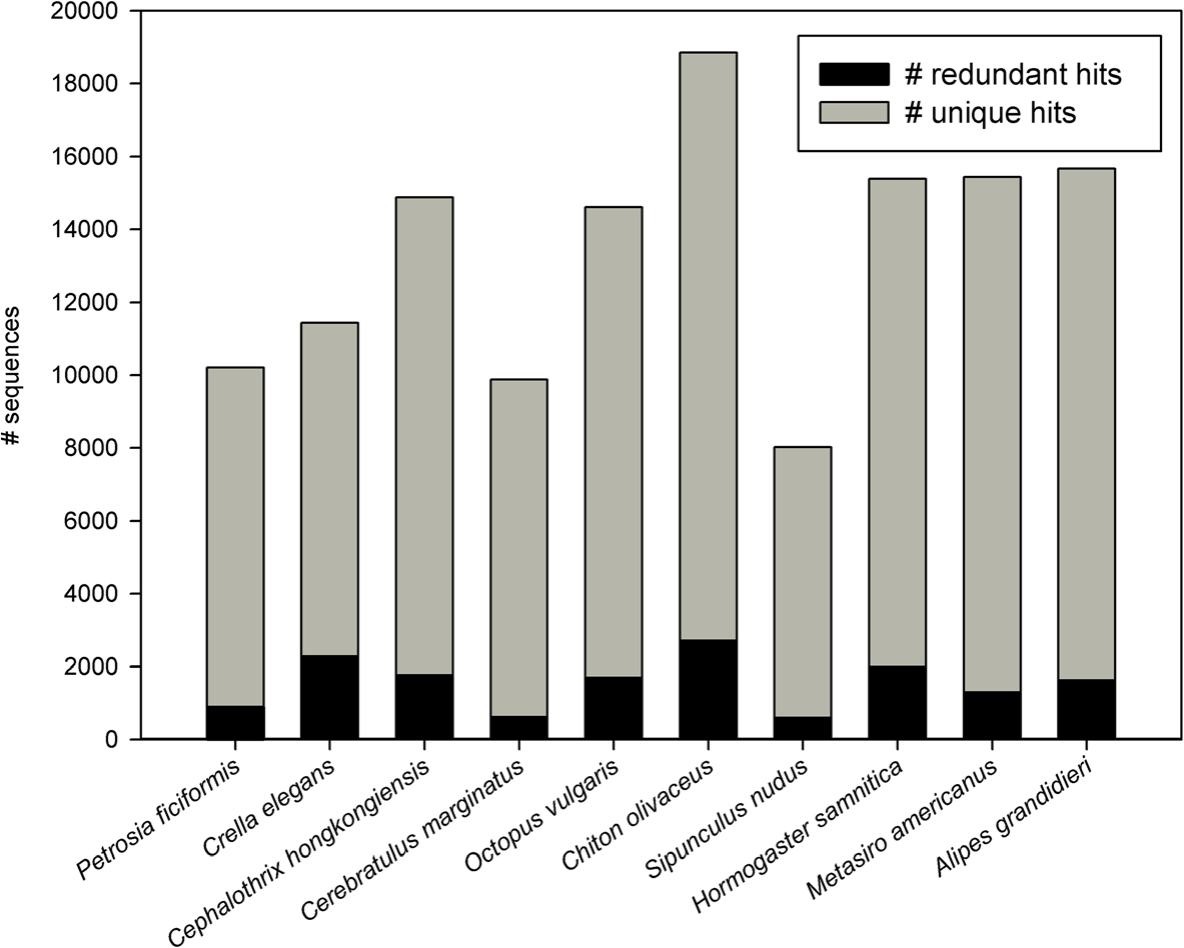
Number of sequences that resulted in unique hits (only one contig matching to each protein) or redundant hits (two or more blast hits matching to each protein) for each species.

Following the criteria of Ewen-Campen et al. [41] we performed a search for specific GO terms of the categories “biological process”, “molecular function”, and “cellular component” (see Figure 4 and Additional file 8) in all species, and compared them among members of the same phylum (in the case of Annelida, between *S*. *nudus* and *H*. *samnitica*). The GO assignment revealed that no functional category of gene function was lacking in any of our transcriptomes. Irrespective of how many sequences were used for the GO assignment (which ranged from 9,069 to 25,681, see Table 4), the percentages of sequences mapped to given GO terms were highly similar for all species (Figure 4 and Additional file 8) and comparable to other animal transcriptomes [1,9,41,45,52,53]. However, the total numbers of GO terms retrieved for each transcriptome were very different across species (Additional file 8), suggesting the lack of sampling bias in the distribution of genes in the nr database. Our results reflect the comparability of the NGS datasets and the pipelines used for their annotation, in spite of intrinsic differences between various assembly strategies.

Detailed comparisons of GOs among our results and other published transcriptome datasets are not easy, because different researchers have focused on GOs relevant to targeted biological questions. For the category “biological process”, we found that around 20% of the sequences grouped under “localization” in all species (Figure 4 and Additional file 8), and more than 10% showed also the categories “gene expression”, “signaling” and “signal transmission” (Figure 4). For “molecular function”, more than 50% of the sequences in every species fell under the “catalytic activity” category (ranging between 2,462 for *Sipunculus nudus* and 6,068 for *C*. *olivaceus*; Additional file 7). Also, “hydrolase activity” contained more than 20% of the sequences in all species (Figure 4 and Additional file 8). For “cellular component”, most sequences belonged to “cytoplasm” (>20%) and “nucleus” (>10%), with very few sequences grouping under “ribosome” (Figure 4 and Additional file 8). Similar results were reported for the categories “molecular function” and “cellular component” in the arthropods *Oncopeltus fasciatus*[41] and *Parhyale hawaiensis*[52], however the most abundant nodes for those arthropods in “biological process” were “gene expression”, “developmental process”, “multicellular organismal development” and “anatomical structure development” (>20%). The over-representation of development-related categories could be the consequence of the use of embryonic tissues for generating transcriptomes, which was the purpose of those studies. This was generally not the case for the species used in this study, excepting *Metasiro americanus*, for which both adults and various juvenile stages were pooled to facilitate comparison with a separate transcriptome of Opiliones that we generated for developmental applications [57,58]. *Apropos*, the *Metasiro* transcriptome had a higher number of GOs for embryonic development than the other 9 transcriptomes (Figure 4). *Octopus vulgaris* also showed a high percentage of GOs for embryonic development (Figure 4), even though in this case only a piece of an arm was used for the extraction. Also, *Chiton olivaceus* showed many sequences with GO associated term for the category “developmental process” (under “biological process”) (Figure 4), and also in this case we did not detect any reproductive tissue prior to homogenization. This could be due to a better annotation of molluscan developmental proteins to which the contigs blasted in this species, given that during the adulthood of some groups, there is a certain level of expression of embryonic and developmental proteins.

For many characterized transcriptomes, among the most abundant categories in “biological function” are “metabolic” and “establishment of localization” processes [43,45,47,48,52]. The category “establishment of localization” was also abundant in our datasets (between 16.5 and 21.7%), with similar results for “metabolic processes” (Figure 4 and Additional file 8; not shown for “metabolic process”). All gene ontology assignments on transcriptomic data (including ours, see Figure 4 and Additional file 8) provided similar results for the categories “molecular function” and “cellular component”, wherein “catalytic (and mainly hydrolase) activity”, and “cytoplasm” and “nucleus” contained the majority of the sequences with assigned GO terms [4,39,43,45,47,48,52,59,60].

#### Protein families

**Figure 5:**
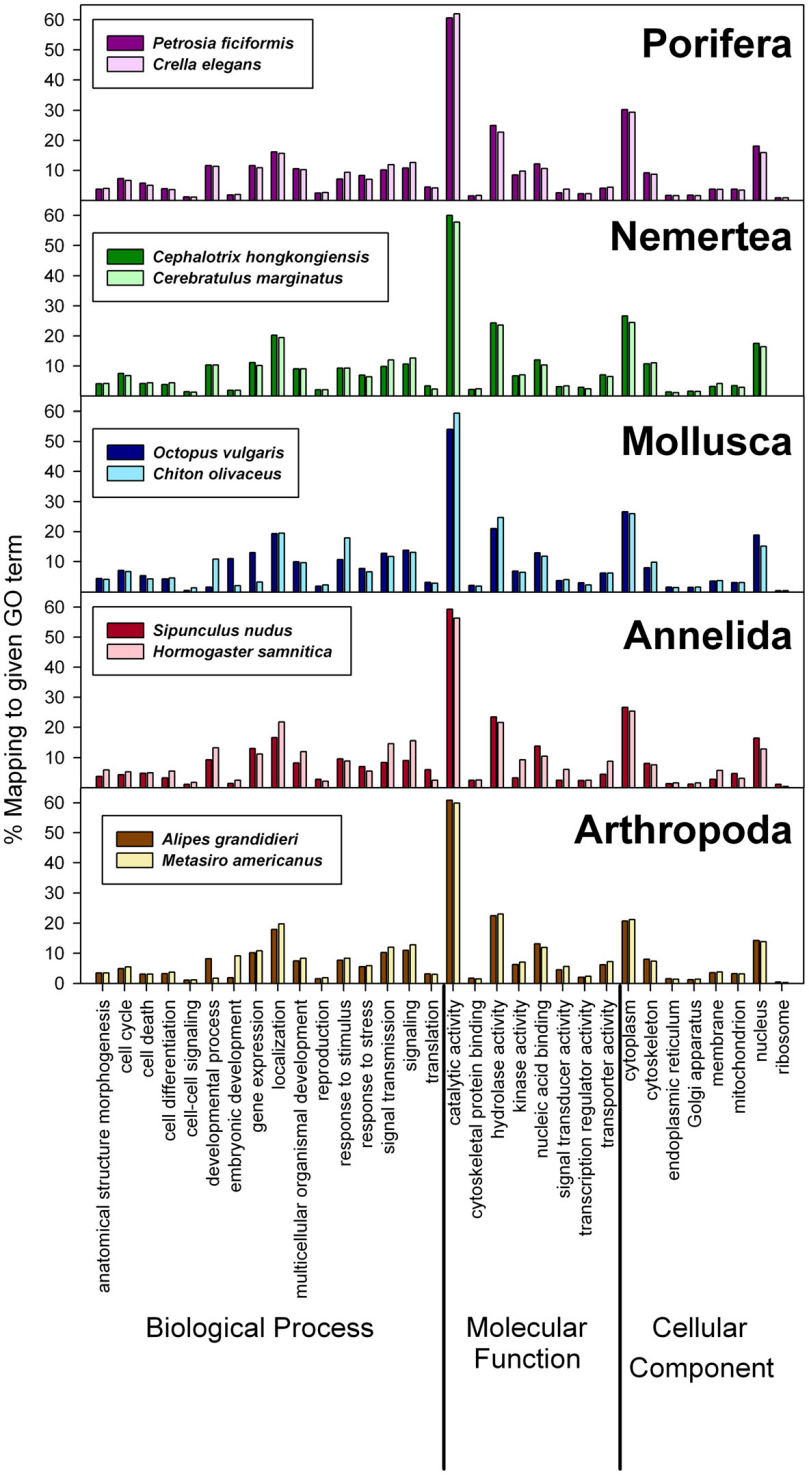
Paired comparison per phylum of the percentages of sequences mapped to given gene ontology (GO) terms.

Searching for conserved domains in the Pfam database showed that ankyrin, WD40, protein kinase, calcium-binding EGF domain, and fibronectin type III domain containing proteins were among the most abundant protein families in all species (Figure 5), as found for other invertebrate transcriptomes [59]. The most abundant protein families in our transcriptomes are known to be involved in integration of cells into tissues, cell adhesion, signal transduction and transcription regulation to cell cycle control, autophagy and apoptosis.

Some protein families, such as those containing death domains, scavenger receptor cysteine-rich domains, and NHL repeats, were very abundant in sponges, whereas in bilaterians they were represented in much lower numbers (Figure 5). In contrast, other protein families (e.g., zinc finger Cys2His2-like proteins, trypsins, and C-type lectins) appear in much higher numbers in bilaterians than in sponges (Figure 5). In our Pfam searches, the MAM domain [61], which is present in proteins like neuropilin, meprin or zonadhesins, was found only in our bilaterian transcriptomes but not in the sponges, and was particularly abundant in *Chiton olivaceus* and *Sipunculus nudus* (Figure 5).

While we found around 550 protein kinases in sponges, the *Amphimedon* genome includes 705 kinases, representing the largest metazoan kinome [62]. Between 380 and 580 protein kinases were also found for both nemerteans, both molluscs, and both arthropods (Figure 5), which constitute higher numbers than those observed for the protein kinase family in the genomes of *Nematostella vectensis*, *Caenorhabditis elegans*, *Drosophila melanogaster*, *Ciona intestinalis*, or *Homo sapiens*[63,64]. Interestingly, in our annelids we found another extreme case, the lowest expressed protein kinase repertoire found in *Sipunculus nudus*, whereas the oligochaete *Hormogaster samnitica* contained more than one thousand protein kinases (Figure 5).

### Estimation of transcriptome completeness

#### Local blast

Transcriptomic datasets can be used as a resource for functional gene screenings or to identify new phylogenetic markers in poorly known organisms. Here, we defined 28 genes belonging to four different categories (the Notch, transforming growth factor β [TGF-β], and Hedgehog signaling pathways; and 7 housekeeping proteins; see details in Table 6) and searched the transcriptome datasets for homologs of each gene. To engender comparability with fully sequenced and annotated invertebrate genomes, we isolated the counterparts of these 28 genes from the complete genomes of *Amphimedon queensladica*[62], *Lottia gigantea* (JGI), and *Capitella teleta* (JGI) using tblastn.

**Table 6:** Individual searches for our transcriptome datasets (no background color) and the JGI genomes of a sponge (pink), a mollusk (violet), and an annelid (green)

	**Notch**										
	**Notch**	**Delta**	**Jagged/ Serrate**	**Fringe**	**HES**	**SuH**	**Deltex**				
***Petrosia ficiformis***	578	247	680	346	234	510	157				
***Crella elegans***	472	247	247	307	85	300	147				
***Amphimedon queenslandica***	1667/614	539	1320	413/370/279	290	656	454				
***Cephalotrix hogkongiensis***	272/173/103	139	-	137	343/309/110	205	168/89				
***Cerebratulus marginatus***	286/495	101	131	140	358/109/167	271	233				
***Chiton olivaceus***	498	358	-	123	289/174/96	168	65				
***Octopus vulgaris***	916	217	-	-	324/247	445	101				
***Lottia gigantea***	2404	724	1245	350	231	549	230				
***Sipunculus nudus***	451/232/241	-	-	-	-	-	-				
***Hormogaster samnitica***	464/546/684/456	521/268/260/388/314/170	-	200/197	350/314/238/173/133/108	521/482	171/952				
***Capitella teleta***	2580/2612/2673/2985	785	1204	207	307/199/141	459/445	610				
***Metasiro americanus***	600	780	552	340	58/179/ 344	493	-				
***Alipes grandidieri***	196/203	151	115/416	66/82	80/294	273	415				
	**TGF**-**β**										
	**TGF-β1**	**Activin**	**Smad 1**	**Smad 2**	**Smad 3**	**dpp**	**BMP1**	**BMP3**	**BMP5**	**BMP6**	
***Petrosia ficiformis***	435	-	230	-	186	-	184	-	-	102	
***Crella elegans***	90	-	408	98	190	-	150	-	-	140	
***Amphimedon queenslandica***	371	-	408/412/181	-	-	-	1035	-	-	413	
***Cephalotrix hogkongiensis***	251	118	151	-	307	-	208	-	114	-	
***Cerebratulus marginatus***	413	120	473	-	266	-	172	-	114	-	
***Chiton olivaceus***	109	285	77	-	242	181	110	-	152		
***Octopus vulgaris***	-	500	302	-	-	97	107	308	-	679	
***Lottia gigantea***	516	523/576	466	428	-	406	332	381	104	-	
***Sipunculus nudus***	-	59	-	-	-	-	153	-	-	-	
***Hormogaster samnitica***	446/317	192	472	-	417	104	226	96	-	-	
***Capitella teleta***	511	471/429	309		452	339	1	239	-	-	
***Metasiro americanus***	362/374	407/71	-	287	-	351	340/613	117	411	160	
***Alipes grandidieri***	425/507	-	117	-	94	173	113	480	-	-	
	**Hedgehog**				**Housekeeping genes**						
	**Hedgehog/ Hedgling**	**Patched**	**Smoothened/ Frizzled**	**Ci/Gli**	**TPI**	**ATPB**	**MAT**	**PFK**	**FBA**	**EF-1α**	**CAT**
***Petrosia ficiformis***	1212	-	288/252/133	343	165	180	94	239	231	253	383/422
***Crella elegans***	327	-	165/156	517	218	267	377/162	139	172/128	460	374
***Amphimedon queenslandica***	1184	-	300/289/275	143	228	184	439/385	840	359	249	508/498
***Cephalotrix hogkongiensis***	-	-	199	-	141	264	-	698	-	191/79	152
***Cerebratulus marginatus***	-	-	100		248	393	100	705	38	331	255
***Chiton olivaceus***	303	-	465	105	235	499	247	421	121	-	190
***Octopus vulgaris***	-	145	590/305/221	-	79	509	332	815	60	109	335
***Lottia gigantea***	355	527	879/572/489	1493	252	521	410	770	273	469	510
***Sipunculus nudus***	91	-	-	-	222	325	247	-	243/113	333	509
***Hormogaster samnitica***	386/301/127	555/536/107	838	695	150	178	402/106	585	213	230/133	458
***Capitella teleta***	329	1465	589/597/591	235	240	479	393	826	364	463	534
***Metasiro americanus***	236	670	75/577	597	236	210	261/182	766	213	207	101
***Alipes grandidieri***	285	132	66	681	235	289	107/78	525	202	124	431

Length of protein sequences are given in amino acids. Abbreviations: *JAG/SER*, jagged and serrate; *HES*, hairy enhancer of split; *Su(H)*, suppressor of hairless; *Dx*, deltex; TGF-β1, transforming growth factor β; *ACV*, activin; Smad, mothers against decapentaplegic; *dpp*, decapentaplegic; *BMP*, bone morphogenetic protein. Asterisks indicate the presence of hedgling instead of hedgehog; *SMO/FZD*, smoothened and frizzled; *Ci/Gli*, cubitus interruptus/*GLI; TPI*, triosephosphate isomerase; *ATPB*, ATP synthase subunit b vesicular; *MAT*, methionine adenosyl transferase; *PFK*, phosphofructokinase; *FBA*, fructose biphosphate aldolase; *EF-1α*, elongation factor-1α; *CAT*, catalase.

Duplications of genes and entire genomes are believed to be important mechanisms underlying morphological variation and functional innovation in the evolution of life, and especially for development of diversity both at a small and a large scale [65-67]. Even though the significance of signaling gene duplications in evolution is not well understood, metazoan phyla demonstrably differ in their number of signaling genes [68]. *In silico* comparisons of the evolution of signaling pathways might reveal then important conclusions. Here, with a very simple approach, we tested the sampling of our transcriptomes for detection of important signaling molecules and their possible duplications in species with limited availability of other genetic resources. For instance, in sponges 100% of the selected genes for the Notch, TGF-β, and Hedgehog signaling pathways that were found in the *A*. *queenslandica* genome were also found in our transcriptomes of *P*. *ficiformis* and *Crella elegans* (Table 6). Our datasets even found gene transcripts in *P*. *ficiformis* (*mothersagainstdecapentaplegic*-1) and in *Crella elegans* (*mothersagainstdecapentaplegic*-1 and *mothersagainstdecapentaplegic*-2) not recovered for *A*. *queenslandica* (Table 6) in our searches or in the genome characterization [62].

Likewise, a high percentage of genes for the Notch, TGF-β, and Hedgehog signaling pathways were found both in the *Lottia* genome and the transcriptomes of our nemerteans and mollusks, with very few absences in each case (see Table 6). Duplication of genes in nemerteans was detected in *notch*, *hairy*/*enhancer*-*of*-*split* (*HES*), and *deltex* (Table 6); while in mollusks gene duplication was found only for *HES*, with three paralogues in *C*. *olivaceus*, and two in *O*. *vulgaris* (Table 6), and *frizzled*, with two paralogues in *O*. *vulgaris* (Table 6). The comparisons between the results obtained for our transcriptomes and the reference genomes of annelids and arthropods were very similar (Table 6). However, the data for *S*. *nudus* were markedly different, as very few genes were recovered from the transcriptome, mainly due to the high redundancy observed in the transcripts.

Other studies with arthropods have taken the same approach, searching for signaling pathway genes in their transcriptome datasets in comparison to reference genomes (e.g. [41,52]). Those cases corroborate comparability between the transcriptomic and the genomic data we observed, although, as in our case, the sequences recovered from the transcriptomes were shorter than the genomic ones. Nevertheless, many of these transcripts are sufficiently long that they can be readily used for phylogenetic inference as well as experimental applications such as *in situ* hybridization and RNAi-mediated gene knockdown (a fragment *ca*. 500 bp in length is sufficient for either of these techniques [52,57,58]).

Genome or gene duplication engender orthologues and paralogues, which have their own evolutionary histories, owing to paralog losses, subfunctionalization, and/or neofunctionalization [65,66,69,70]. Failure to detect paralogues can lead to misinterpretations of cellular biochemistry, and often inaccuracies in reconstructions of phylogeny and molecular evolution [71,72]. Here, transcriptome sequencing proved to be useful in paralogue detection, for which traditional methods (e.g., cloning and colony PCR) are inefficient. All housekeeping genes were found among our transcriptomes, barring a few absences (see Table 6), with very similar results also found in the selected genomes. However, the most interesting results involved the paralogues found for four housekeeping genes. The poriferans *A*. *queenslandica* and *P*. *ficiformis* (both constituents of the order Haplosclerida) have two paralogues for *catalase* (CAT; Table 6) of ca. 400 amino acids in length. The gene *fructose biphosphate aldolase* (*FBA*) has also two paralogues in *Crella elegans* and *S*. *nudus* (Table 6). The nemertean *C*. *hongkongiensis* and the annelid *H*. *samnitica* each have two paralogues for *elongation factor1α* (*EF1α*) (Table 6). Two or three paralogues were found for all species for the gene *elongation factor thermo unstable* (*EFTu*; not shown in Table 6) which contains a very similar domain to *EF1α* and is localized in the mitochondria [73]. *Methionine adenosyltransferase* (*MAT*) has two paralogues in the sponges *A*. *queenslandica* and *Crella elegans*, in the earthworm *H*. *samnitica*, and in the arthropods *M*. *americanus* and *A*. *grandidieri* (Table 6).

**Figure 6:**
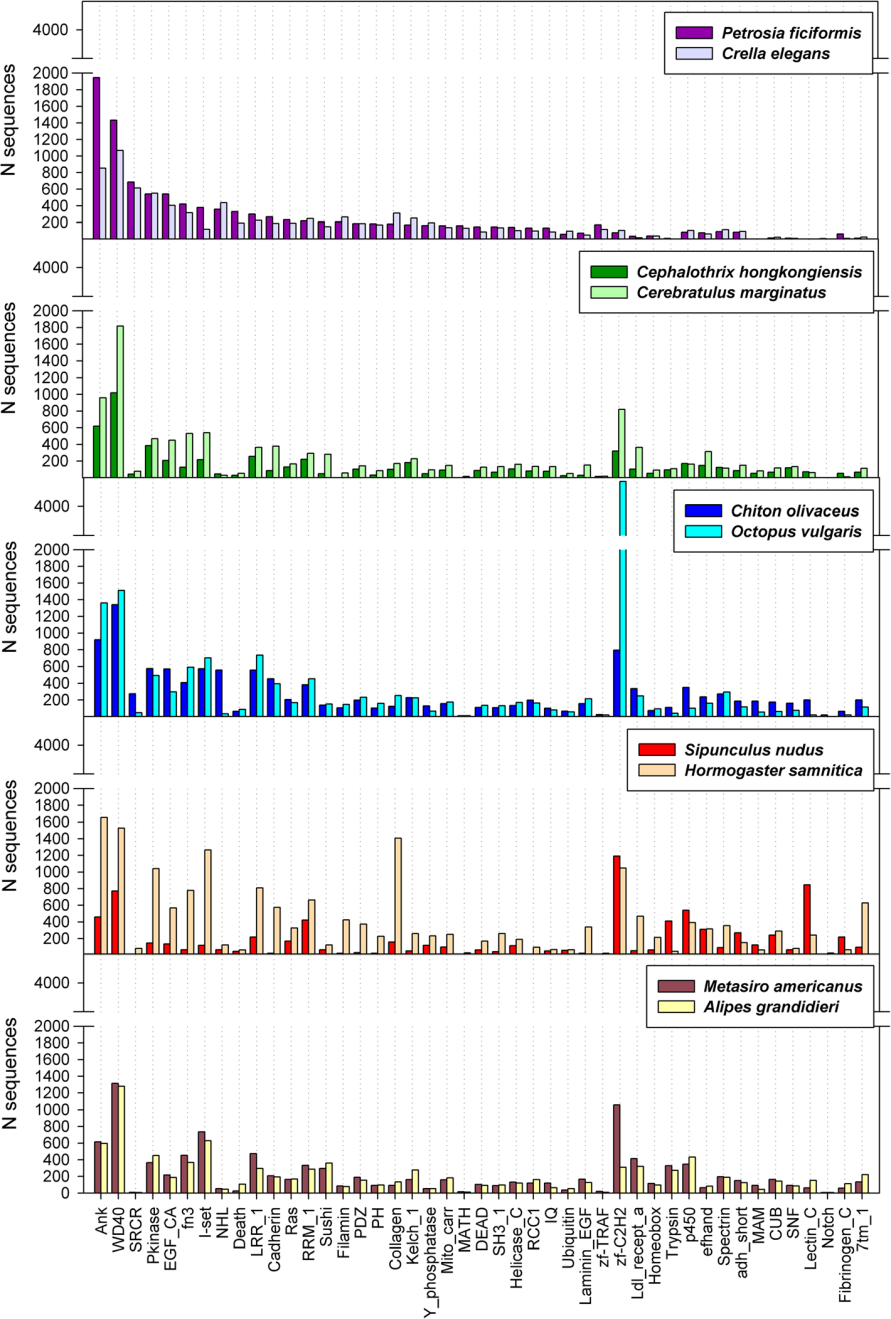
Compared abundances of PFAM domains for selected domains.

Housekeeping genes are frequently used as phylogenetic markers because they are putatively paralogy-free [72]. According to our survey of housekeeping genes, at least five are shown to have two or more paralogues. In order to test whether they bear similar or contradicting phylogenetic signals, we constructed a phylogenetic tree using all paralogues we found in our transcriptomes for the gene *MAT* (Figure 6). While the paralogues of *C*. *elegans* and *H*. *samnitica* clustered, neither the two paralogues of *M*. *americanus*, nor those of another Opiliones, *Phalangium opilio*, formed a clade, suggesting the possibility of ancient duplications of *MAT* in chelicerate arthropods. Thus, the use of each paralogue sequence for phylogenetic purposes needs to be carefully evaluated, as ignorance of paralogy or erroneous assumption of single-copy genes can confound inference of tree topology. This might be the case for several arthropod phylogenies, which were constructed using genes afflicted by paralogy. For example, in centipedes (Arthropoda, Myriapoda, Chilopoda), it was previously observed that datasets dominated by nuclear ribosomal genes favored one topology that accorded greatly with morphological and paleontological data [74,75]. By contrast, datasets comprised of three nuclear protein-encoding genes (*elongation factor1α*, *elongation factor2*, and *RNA polymerase II*) favored a radically different topology, with a derived placement of the lineage traditionally considered sister to the remaining centipedes [76]. It was shown that this conflict originated in the nuclear coding markers [74,77], and a subsequent phylogenomic analysis using 62 protein-coding genes [78] vindicated the traditional phylogeny of the group (sensu [79]). This was also the case for the arthropod *M*. *americanus*, in which direct sequencing of clones *for elongation factor1α* revealed numerous and non-concerted paralogous copies of *elongation factor1α* (as in *MAT*, above), hindering use of this marker in studies of statistical phylogeography [80]. It is possible that conflicts documented between ribosomal and protein-encoding data partitions in arthropod (and other) phylogenies are attributable to paralogy in one or both types of data. In addition to refining phylogeny analysis, recognition of paralogy will improve our understanding of the evolutionary processes that generated biochemical, cellular, and developmental innovations [70].

#### Ortholog hit ratio

**Figure 7:**
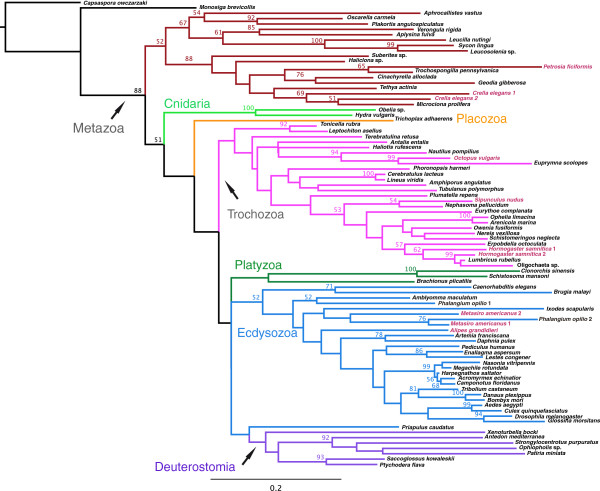
**Phylogenetic reconstruction of metazoans using the gene methionine adenosyl transferase.** Only bootstrap support values above 50% shown. Sequences derived from our transcriptomes are shown in red. GenBank accession numbers for all sequences used can be found in Additional file 9.

**Figure 8:**
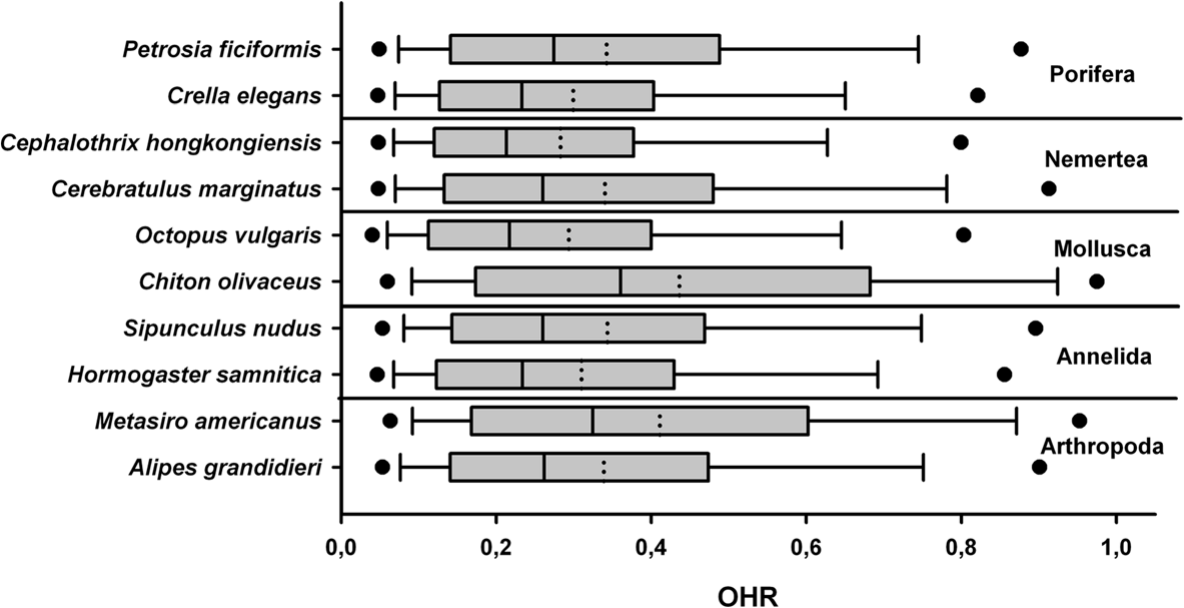
Ortholog hit ratio (OHR) analysis showing the median (solid line), the mean (dotted line) and the 95^th^ and 5^th^ percentiles for all species.

The ortholog hit ratio (OHR) is an estimate of the amount of a transcript contained in a gene, with respect to a reference sequence. Ortholog hit ratios greater than 1.0 likely indicate large insertions in genes [60]. It is important to note that to calculate the OHR, we used as reference the first blast hit for each of the contigs; final OHR estimation is a function of the completeness of those references, which in many cases were partial sequences. Given the phylogenetic distances between some of the taxa sequenced here and those for which full genomes are currently available, one of our outstanding concerns was that the OHR would be higher for certain taxa as an artifact of genomic resource availability. We anticipated that the OHR of the arthropods, for which many genomes are available, would be especially affected. However, we observed that the average values for the OHR in all our species were around 0.3 (Figure 7 and Additional file 9), similar to OHR values of the organisms in which OHR had been previously calculated (all arthropods [41,52,81]). Given that sequences were obtained with short read transcriptomic data, it was expected that the length of the sequence would be inversely proportional to OHR (Figure 7 and Additional file 9). We did not observe significant differences between the medians or quartiles of the OHR across our taxa (Figure 8). It may be that the quality of the RNA extraction, and also an unbiased mRNA fragmentation, may be better predictors of the mean OHR than the phylogenetic affinity of the focal taxon, although this prediction was not tested in our study. These data suggest that in the future, as complete genomes are obtained for all animal phyla, the OHR values presently obtained might change, but in a manner irrespective of phylogenetic affinity.

### Reassembly of datasets

**Figure 9:**
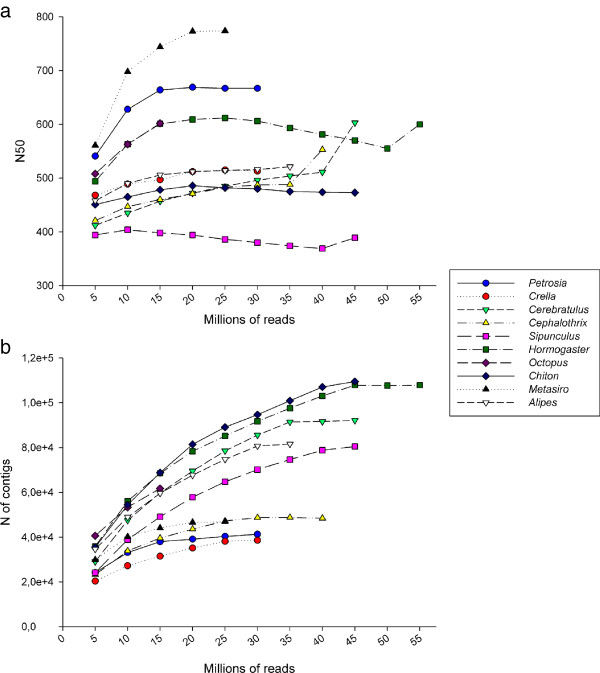
**Assembly of the transcriptome datasets through sequential addition of 5 million reads.a**: N50; and **b**: total number of contigs, were plotted against the different assemblies obtained for each species. Note that the final values in this figure are different from those in Table 2 because we used a newer version of CLC Genomics Workbench (v. 5.1).

We assessed the completeness of the datasets by reassembling all datasets, adding 5 million reads per iteration. Following this approach, number of contigs for most transcriptomes had saturated by the time the 5 million reads where added (Figure 9), except for *S*. *nudus* and *O.vulgaris*. For the N50, only *O*. *vulgaris*, *C*. *hongkongiensis*, *C*. *marginatus*, and *H*. *samnitica* increased slightly their values when adding the last batch of reads. With this analysis, we accrue confidence that sequencing efforts were sufficient to estimate accurately the completeness of our transcriptomic datasets (excepting *S*. *nudus*, which had other limitations in data quality and assembly). It is important to note that the assembly statistics obtained during reassembly were not strictly in concordance with those obtained in the first *de novo* assembly for the datasets, as a newer version of the software was used in this case (CLC Genomics Workbench 5.1).

## Conclusions

Reduction in sequencing costs and the unprecedented amount of data facilitated by NGS foretells access to a plethora of biological applications in many disciplines, and provides genetic resources essential for expanding understanding of comparative organismal biology and evolutionary history. Here we generated comparative transcriptomic data for ten non-model invertebrates in multiple phyla (Annelida, Arthropoda, Mollusca, Nemertea, and Porifera) using the Illumina sequencing platform, and produced a tractable catalogue of raw contig sequences and annotated genes for application in phylogenetic analysis, gene expression profiling, and/or developmental analysis. The identity of the lineage and genomic resources previously available for each phylum did not affect metrics of assembly quality. Gene Ontology assignments indicated that no functional gene category was absent or insufficiently sampled in any of the transcriptomes, corroborating the consistency of our pipelines with regard to sequencing and depth of annotation. Finally, we found that our datasets are a useful resource for paralogue detection.

## Methods

### Sample collection

We collected tissue samples from 10 invertebrate species, belonging to five phyla, Annelida (including Sipuncula), Arthropoda, Mollusca, Nemertea, and Porifera, (Figure 1), which include members of several major animal clades [82]. Collecting information is provided in Table 1.

### Sample preparation

For sponge and earthworm samples, in order to avoid contaminations from epibionts, tissues were carefully cleaned (and the gut removed in the earthworm) using a stereomicroscope. Tissue excisions were always performed with sterilized razor blades rinsed in RNAseZap® (Ambion, Texas, US). All cleaning procedures were operated as quickly as possible to avoid RNA degeneration in an RNAse-free and cold environment (in dishes kept on ice, for example).

Preservation of tissues was performed soon after the animals were collected, usually 1 to 5 hours later depending on the time required for cleaning samples. Tissues were cut into pieces from 0.25 cm to 0.5 cm in thickness, except for tissues of *C*. *hongkongiensis*, which were not chopped due to small size. Usually, between 20 to 80 mg of tissue were placed in each eppendorf tube for subsequent processing. Tissue samples were either flash-frozen in liquid nitrogen and immediately stored at −80°C; or they were immersed in at least 10 volumes of RNA*later*® at 4°C for 1 hour, incubated overnight at −20°C, and subsequently stored in the same buffer at −80°C until RNA was extracted (sometimes samples placed in RNA*later* were transported back to the lab at room temperature, and then stored at −80°C).

### mRNA extractions

Two different methods of RNA extraction were used: 1) total RNA extraction followed by mRNA purification for nemerteans, molluscs, annelids, and arthropods, and 2) direct mRNA extraction for sponges. Protocols used for both extraction types are available elsewhere [83].

#### Quantity and quality control of mRNA

Quantity and quality (purity and integrity) of mRNA were assessed by three different methods. We measured the absorbance at different wavelengths using a NanoDrop ND-1000 UV spectrophotometer (Thermo Fisher Scientific, Wilmington, Massachusetts, USA). Quantity of mRNA was also assessed with the fluorometric quantitation performed by the QubiT® Fluoremeter (Invitrogen, California, USA). Also, capillary electrophoresis in an RNA Pico 6000 chip was performed using an Agilent Bioanalyzer 2100 System with the “mRNA pico Series II” assay (Agilent Technologies, California, USA). Integrity of mRNA was estimated by the electropherogram profile and lack of rRNA contamination (based on rRNA peaks for 18S and 28S rRNA given by the Bioanalyzer software).

### Next-generation sequencing

Next-generation sequencing was performed using the Illumina GAII platform (Illumina, Inc., San Diego, California, USA) at the FAS Center for Systems Biology at Harvard University. mRNA concentrations between 11.5 and 77.4 ng/μL (Additional file 1) were used for cDNA synthesis, which was performed following methods published elsewhere [83]. cDNA was ligated to homemade adapters (designed by Steve Vollmer, *personal communication*) in *Petrosia ficiformis* (5^′^-ACA CTC TTT CCC TAC ACG ACG CTC TTC CGA TCT GGT T-3') and in *Crella elegans* ( 5^′^-ACA CTC TTT CCC TAC ACG ACG CTC TTC CGA TCT CAG T-3') whereas ds cDNA was ligated to Illumina adapters in the rest of species. Size-selected cDNA fragments of around 300 bp (Additional file 1) excised from a 2% agarose gel were amplified using Illumina PCR Primers for Paired-End reads (Illumina, Inc.) and 18 cycles of the PCR program 98°C-30 s, 98°C-10 s, 65°C-30 s, 72°C-30 s, followed by an extension step of 5 min at 72°C.

The concentration of the cDNA libraries was measured with the QubiT® dsDNA High Sensitivity (HS) Assay Kit using the QubiT® Fluoremeter (Invitrogen, Carlsbad, California, USA). The quality of the library and size selection were checked using the “HS DNA assay” in a DNA chip for Agilent Bioanalyzer 2100 (Agilent Technologies, California, USA). Four different profiles of cDNA libraries were obtained consistently: 1, a tight band of targeted size with high cDNA concentration; 2, a tight band of targeted size and additional “bumps” of smaller or larger fragments; 3, no bands; 4, a tight band of targeted size with low cDNA concentration. cDNA libraries were considered successful when the final concentration was higher than 1 ng/μL and the Bioanalyzer profile was optimal (1 or 2) [83]. Successful libraries were brought to 10 nM or 7nM depending on the initial concentration prior to sequencing. The paired-end reads had lengths of 101 bp for the sponge, nemertean, annelid, and sipunculan species, and 150 bp for the mollusk and arthropod species.

### Sequence assembly

Removal of low quality reads or portions of them (i.e., thinning and trimming analyses) for the raw reads was done with CLC Genomics Workbench 4.6.1 (CLC bio, Aarhus, Denmark). Thinning refers to discarding of nucleotides and/or entire reads based on quality parameters. It was performed using 0.05 (Assembly A) and 0.005 (Assembly B) as the limit (based on *Phred* quality scores (q) [84], where the q is converted into a probability (p) of error in 10^q/-10^, and the limit – p will be negative when the quality is low). The resulting quality of the thinned reads was visualized FastQC (http://www.bioinformatics.bbsrc.ac.uk/projects/fastqc/). After thinning, only those terminal bases with a Phred quality score under 30 were trimmed (where a *Phred* score of 30 corresponds to a probability of 10^-3^ of incorrect base calling; see Table 2 and Additional file 2), producing sequences of unequal size (i.e., trimming). Reads were re-screened to check for presence of adapter or primer sequences using FastQC, and if present, they were removed using CLC Genomics Workbench 4.6.1.

*De novo* assemblies with all datasets thinned and trimmed with various parameters were performed with CLC Genomics Workbench 4.6.1 (CLC bio, Aarhus, Denmark) using the same protocol. Global alignments for the *de novo* assemblies were always done using the following default parameters: mismatch cost=2; insertion cost=3; deletion cost=3; length fraction=0.5; similarity=0.8; and randomly assigning the non-specific matches. Best *k*-mer length was estimated by the software. The best assembly for each species was selected using an adaptation of the optimality criteria for *de novo* assembly with 454 data (see Additional file 3), [38], being the number of contigs, the mean contig length, the N50, the number of contigs greater than 1 Kb, and the maximum contig length, the most relevant criteria utilized.

### Sequence annotation

For each species, contigs shorter than 300 bp were removed, as very few of these short contigs retrieved results for Gene Ontology assignments. For example, for *Petrosia ficiformis*, 49,246 contigs were shorter than 300 bp, only 22.3% returning blast hits, and only 1.5% of them returning a Gene Ontology ID. The remainder contigs were mapped against a selection of the non-redundant (*nr*) NCBI database (only proteins of Metazoa and Fungi) using the blastx program of the BLAST suite. All searches were conducted with Blastall [85,86] using an e-value cut-off of 1e-5. With the resulting file, we then used Blast2GO v2.5.0 [87] to retrieve the Gene Ontology (GO) terms and their parents associated with the top 20 BLAST hits for each sequence. Also, using Interproscan tools (http://www.ebi.ac.uk/Tools/pfa/iprscan/), the hidden Markov models (HMMs) that are present in the PFAM Protein families database were recovered.

### Estimating sequence depth

To estimate the complexity of the resulting assemblies independently from the general blast results, we selected gene targets from conserved developmental signaling pathways and also genes commonly used for phylogenetic purposes (Table 6). We downloaded three different orthologues of the selected protein targets from several invertebrate species (trying to cover the animal phylogenetic span), and searched them in our transcriptomes (using the tblastn engine implemented in CLC Genomics Workbench 4.6.1). We selected only the hits with the maximum similarity (which varied greatly between groups), and checked each open reading frame with ORF finder (http://www.ncbi.nlm.nih.gov/gorf/orfig.cgi). Each predicted protein sequence was re-blasted against the database nr in NCBI using the blastp program (http://blast.ncbi.nlm.nih.gov/) and the domain structure rechecked with SMART (http://smart.embl-heidelberg.de/) using HMMER, PFAM domain, and internal repeats searching. If two independent genes blasted (in the re-blasting) against the same protein of a metazoan that could not be considered an epibiont or symbiont but most likely our sequenced species, we considered them tentative paralogues. These tentative paralogues were aligned with SEAVIEW 4.3.0 [88] and only those with overlapping regions were taken into account. Then, pairwise comparisons were performed between all the paralogues for the same gene, and only those showing more than 20 percent of identity were used. We used the genomes of *Amphimedon queenslandica*, *Lottia gigantea*, and *Capitella capitata* (available in JGI: http://genome.jgi.doe.gov/genome-projects/) to compare the results obtained using the same strategy searching for the selected genes.

We also estimated the ortholog hit ratio (OHR), as defined by O’Neil et al. [60], which describes the percentage of an ortholog found in a contig by dividing the number of non-gap characters in the query hit by the length of the subject, using the script of Ewen-Campen et al. [41]. The workflow used to analyze all our transcriptomic data is shown in Figure 2.

In addition, to analyze the level of completeness of our datasets (since no reference genome is available for the species selcected), we divided the original sequence files (raw reads) in smaller files containing 5 million reads each, and reassembled all the transcriptomes adding up a file each time. We then measured the number of contigs and N50 for each sequential assembly.

### Phylogenetic analysis

The discovery of multiple paralogues for several housekeeping genes, which were putatively in single-copy, encouraged us to test whether the different paralogues bore distinct phylogenetic signals. We selected the gene methionine adenosyltransferase, which showed two paralogues for the sponge *Crella elegans*, the annelid *Hormogaster samnitica*, and the arthropod *Metasiro americanus* (the arthropod *Alipes grandidieri* also had two paralogues for the gene, but one of the transcripts was very short and not suitable for phylogenetic comparisons). Sequences for sponges and arthropods were downloaded from GenBank (Additional file 10) and independent protein alignments were built for sponges and arthropods using SEAVIEW 4.3.0 [88]. Maximum likelihood analysis was conducted using RAxML ver. 7.2.7 [89] on 20 CPUs of a cluster at Harvard University, FAS Research Computing (odyssey.fas.harvard.edu). For the maximum likelihood searches, a unique WAG model of sequence evolution with corrections for a discrete gamma distribution (WAG + Γ) was specified for each data partition, and 500 independent searches were conducted. Nodal support was estimated via the rapid bootstrap algorithm (1000 replicates) using the WAG-CAT model [90]. Bootstrap resampling frequencies were thereafter mapped onto the optimal tree from the independent searches.

## Competing interests

The authors declare that they have no competing interests.

## Authors’ contributions

AR participated in the conception of the study, carried out the molecular genetic studies, coordinated and participated in the analysis, and drafted the manuscript. ARPP, MN, PS, SA, and VV carried out the molecular genetic studies, participated in data processing and analysis, and helped to draft the manuscript. GK and VG carried out the molecular genetic studies and participated in data processing and analysis. GG participated in the conception of the study, its design and coordination, and helped to draft the manuscript. All authors read and approved the final manuscript.

## Supplementary Material

Additional file 1 Details of mRNA concentration, cDNA concentration, library quality, and fragment size of the sequenced fraction of the library for each studied species. Click here for file

Additional file 2 **Assembly parameters for all assembly trials per species.** Thinning was performed using 0.05 (Assembly A) and 0.005 (Assembly B) as the limit in CLC Genomics Workbench. The number of bases removed from the 3′ end after trimming is indicated. Selected assemblies are shown in orange. Abbreviations: N, number; BT, before thinning and trimming; AT, after thinning and trimming; Mb, megabases; bp, base pairs; avg., average; L, length; SD, standard deviation. Click here for file

Additional file 3 Correlation between read length after trimming in base pairs (bp) and the maximum contig length in bp obtained for each assembly. Click here for file

Additional file 4 Optimality criteria for the selection of best assembly. Click here for file

Additional file 5 Coverage values for each transcriptome dataset. Click here for file

Additional file 6 **Contigs over 1Kb for each species and the respective percentage (%) of the total number of contigs.** N: number. Click here for file

Additional file 7 Percentage of contigs showing no blast hit (none), blast hits against the NCBI database nr (blast), and Gene Ontology assignments (annot) for each species. Click here for file

Additional file 8 Number of sequences with Gene Ontology (GO) assignment for defined functional categories in each species. Click here for file

Additional file 9 Plot of the Ortholog Hit Ratio (OHR) for each species. Note the logarithmic nature of the Reference length (x-axis). Click here for file

Additional file 10 Accession numbers of GenBank (regular font) and Short Read Archive (SRA; in bold letters) for amino acid sequences of the protein Methionine adenosyltransferase. Click here for file
