# Supplementary figures and images for: Comparative description of ten transcriptomes of newly sequenced invertebrates and efficiency estimation of genomic sampling in non-model taxa
Source: Front Zool. 2012 Nov 29;9:33. doi: 10.1186/1742-9994-9-33 (PMC3538665; doi:10.1186/1742-9994-9-33)

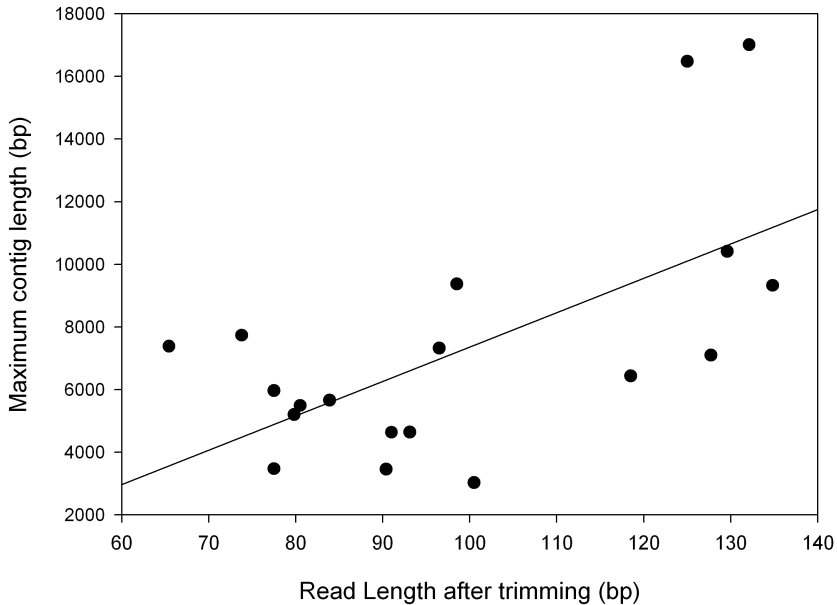

Supplement: Additional file 3 — Correlation between read length after trimming in base pairs (bp) and the maximum contig length in bp obtained for each assembly. [file 1742-9994-9-33-S3.pdf]

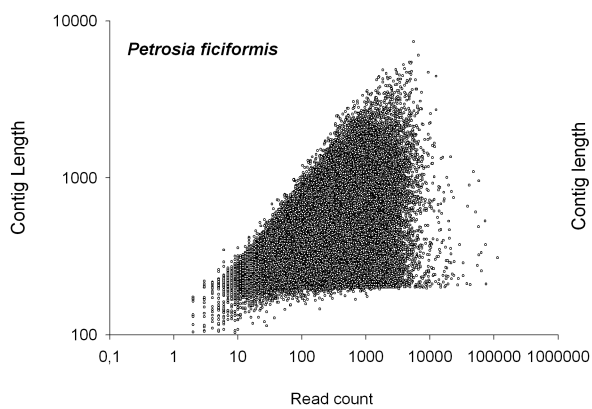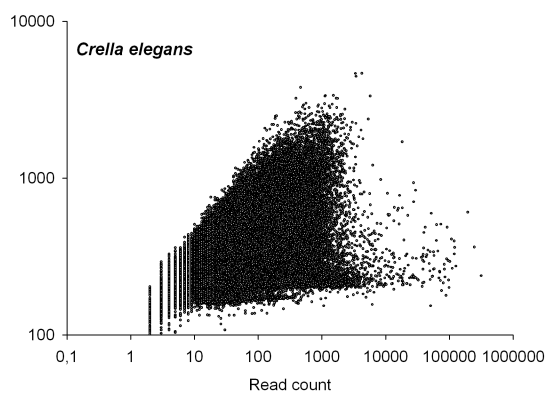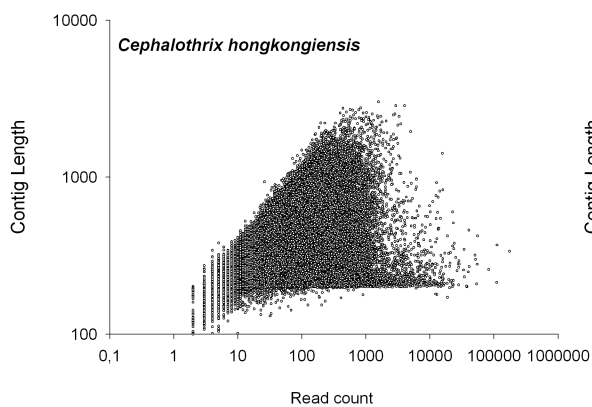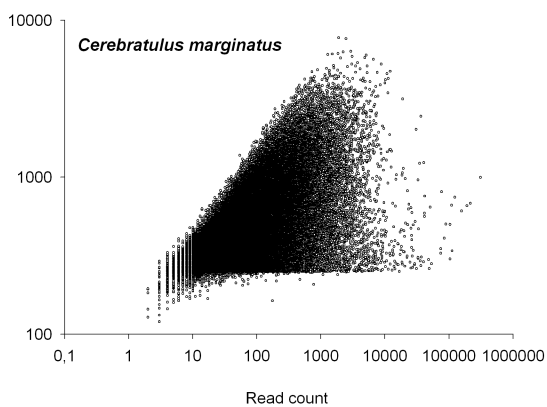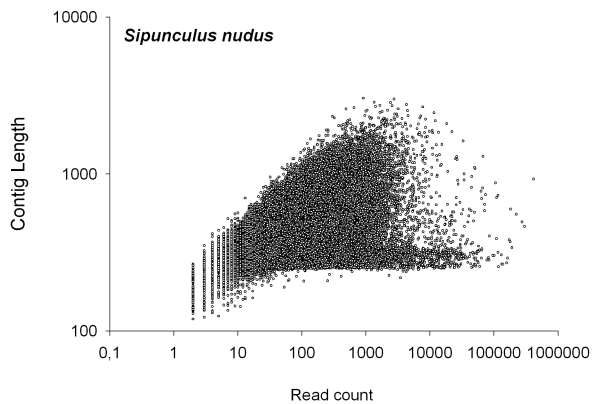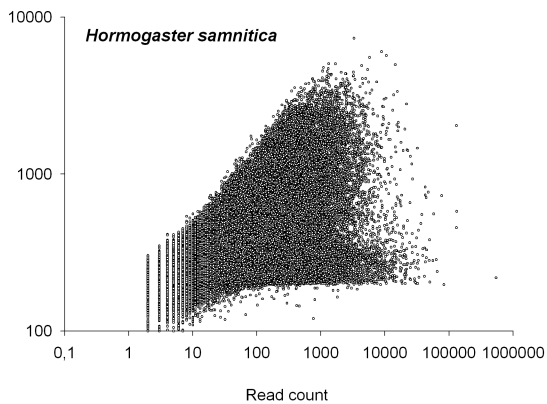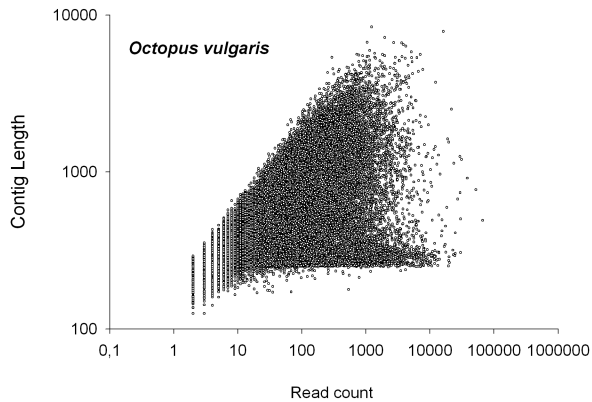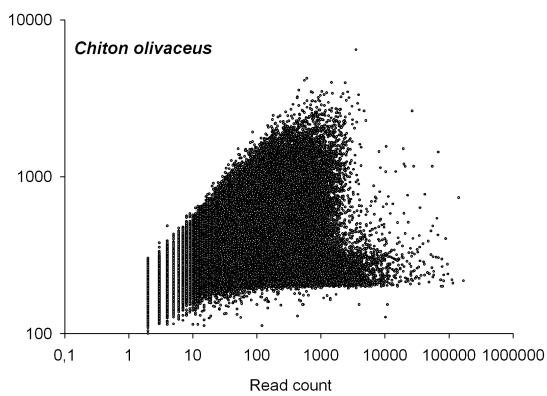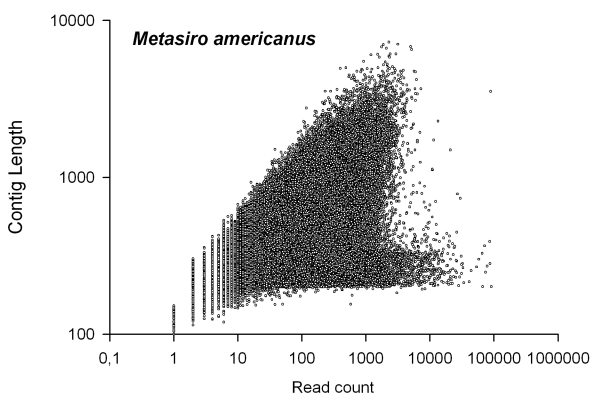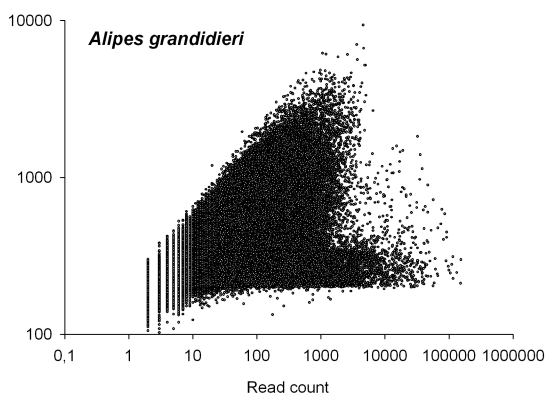

Supplement: Additional file 5 — Coverage values for each transcriptome dataset. [file 1742-9994-9-33-S5.pdf]

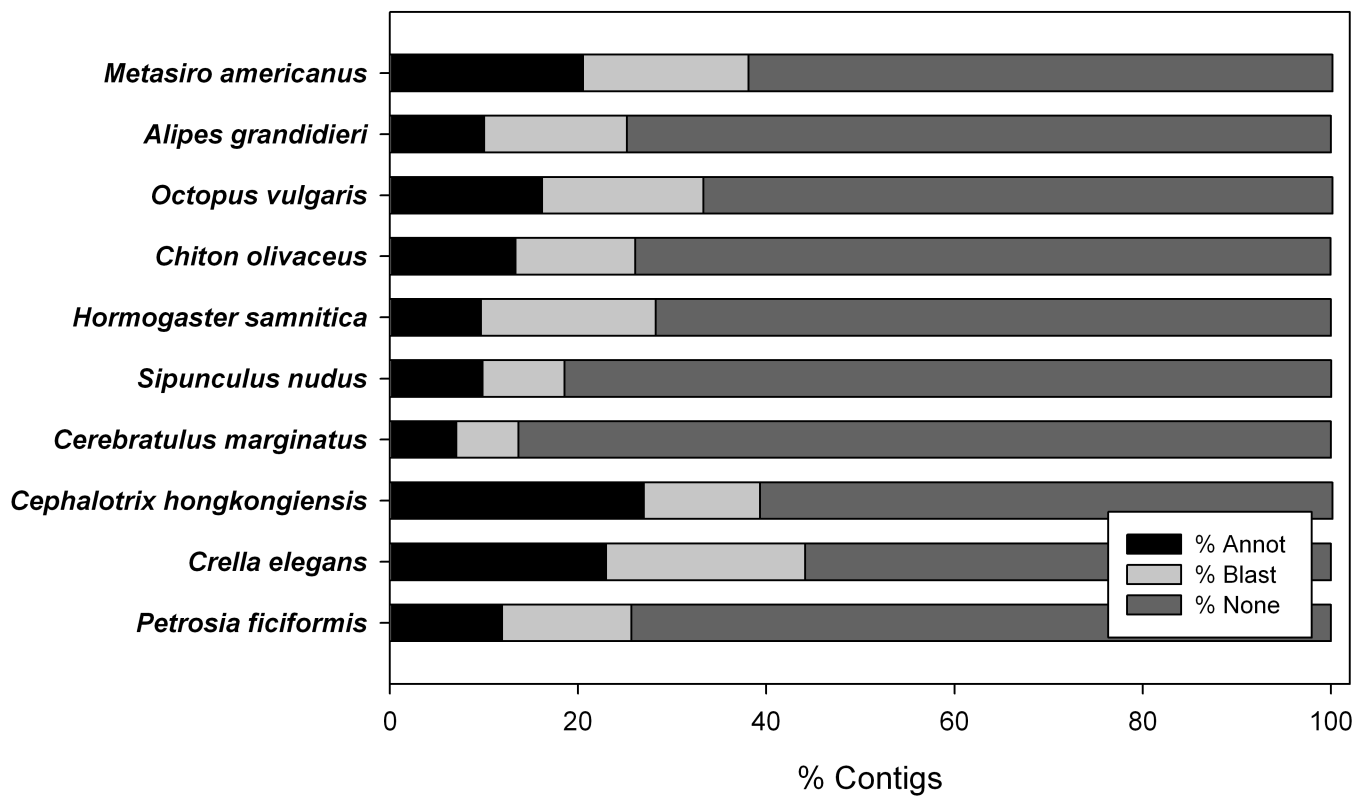

Supplement: Additional file 7 — Percentage of contigs showing no blast hit (none), blast hits against the NCBI database nr (blast), and Gene Ontology assignments (annot) for each species. [file 1742-9994-9-33-S7.pdf]

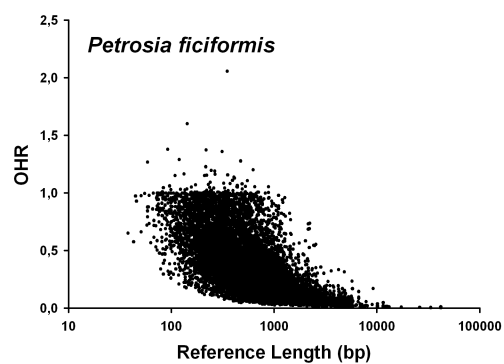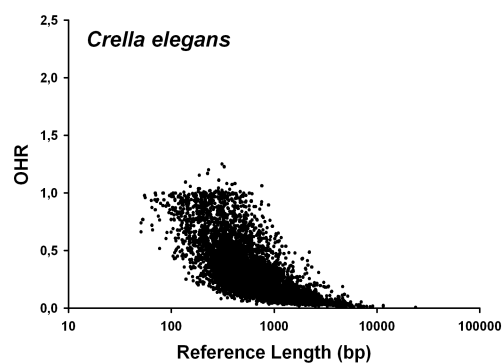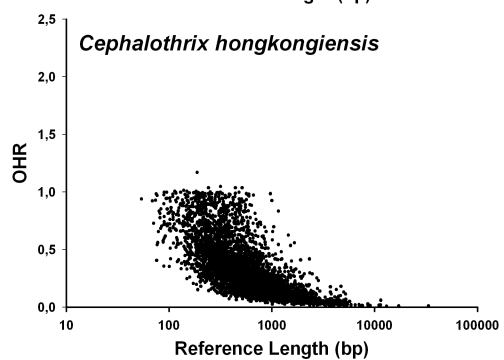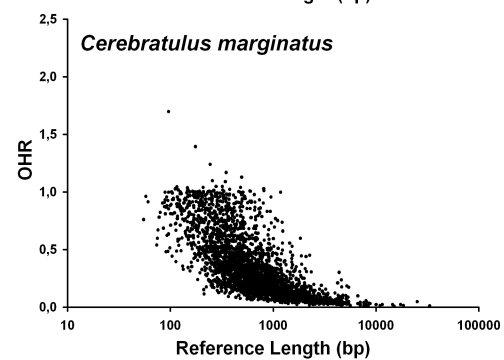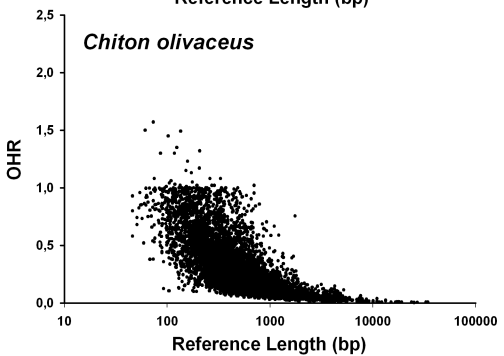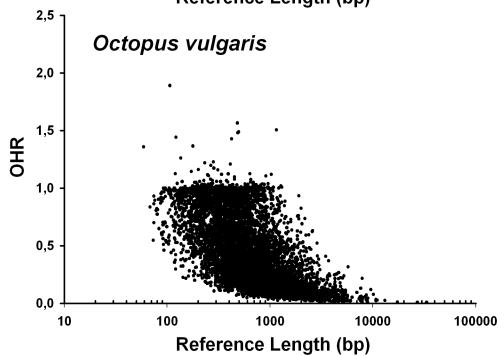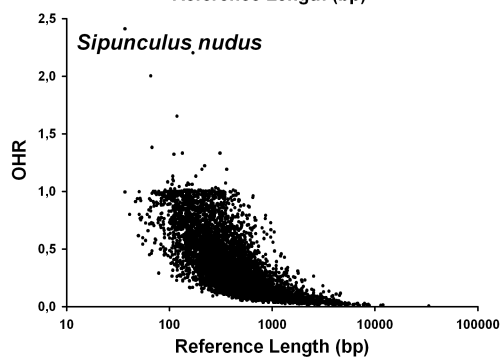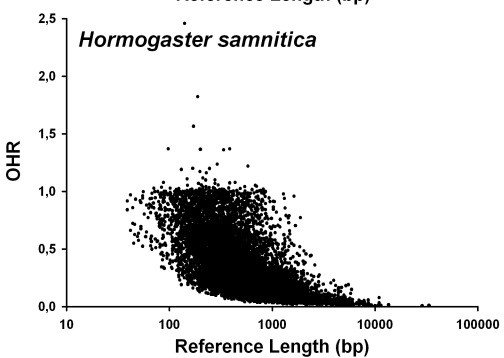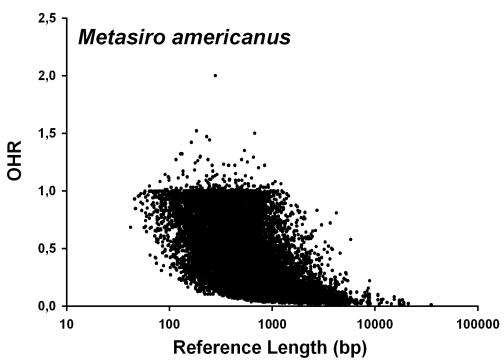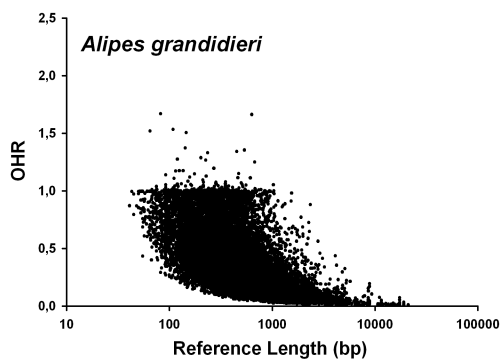

Supplement: Additional file 9 — Plot of the Ortholog Hit Ratio (OHR) for each species. Note the logarithmic nature of the Reference length (x-axis). [file 1742-9994-9-33-S9.pdf]
